# Patients at risk of nontuberculous mycobacterial pulmonary disease who need testing evaluated using a modified Delphi process by European experts

**DOI:** 10.1183/23120541.00791-2023

**Published:** 2024-09-23

**Authors:** Michael R. Loebinger, Stefano Aliberti, Charles Haworth, Mateja Jankovic Makek, Christoph Lange, Natalie Lorent, Apostolos Papavasileiou, Eva Polverino, Gernot Rohde, Nicolas Veziris, Dirk Wagner, Jakko van Ingen

**Affiliations:** 1 Royal Brompton Hospital and NHLI, Imperial College London, London, UK; 2 Department of Biomedical Sciences, Humanitas University, Milan, Italy; 3 Respiratory Unit, IRCCS Humanitas Research Hospital, Milan, Italy; 4 Cambridge Centre for Lung Infection, Royal Papworth Hospital, Cambridge, UK; 5 Department of Medicine, University of Cambridge, Cambridge, UK; 6 Clinic for Respiratory Diseases, University Hospital Center Zagreb, Zagreb, Croatia; 7 University of Zagreb, School of Medicine, Zagreb, Croatia; 8 Division of Clinical Infectious Diseases, Research Center Borstel, Borstel, Germany; 9 German Center for Infection Research (DZIF); 10 Respiratory Medicine and International Health, University of Lübeck, Lübeck, Germany; 11 Global TB Program, Baylor College of Medicine and Texas Children's Hospital, Houston, TX, USA; 12 Department of Respiratory Medicine, University Hospitals Leuven, Leuven, Belgium; 13 Department of Chrometa, Laboratory of Respiratory Diseases and Thoracic Surgery (BREATHE), KU Leuven, Leuven, Belgium; 14 Department of Mycobacterial Infections, Sotiria Athens Hospital of Chest Diseases, Athens, Greece; 15 Pneumology Dept, Hospital Universitari Vall d'Hebron, Vall d'Hebron Institut de Recerca, Vall d'Hebron Barcelona Hospital Campus, Ciber de Enfermedades Respiratorias, Barcelona, Spain; 16 Department of Respiratory Medicine, Goethe University Frankfurt, University Hospital, Frankfurt am Main, Germany; 17 Département de Bactériologie, Sorbonne Université, Centre d'Immunologie et des Maladies Infectieuses (Cimi-Paris), UMR 1135, Hôpital Saint-Antoine, Centre National de Référence des Mycobactéries, APHP, Sorbonne Université, Paris, France; 18 Division of Infectious Diseases, Department of Internal Medicine II, Freiburg University Medical Centre, Freiburg, Germany; 19 Department of Medical Microbiology, Radboud University Medical Center, Nijmegen, the Netherlands

## Abstract

**Background:**

Identifying patients at risk of nontuberculous mycobacterial pulmonary disease (NTM-PD) is challenging. Delays in NTM-PD identification and management are associated with declining lung function and increased morbidity and mortality.

**Study design and methods:**

European NTM-PD experts (n=12) participated in a three-round modified Delphi process to score symptoms and comorbidities potentially associated with NTM-PD as reasons to test for nontuberculous mycobacteria.

**Results:**

Experts reached a consensus on the symptoms and comorbidities that should and should not prompt testing for nontuberculous mycobacteria. Requirements for testing were scored as high (mean ≥7), medium (mean ≥4–<7) or low (mean <4). Nontuberculous mycobacteria testing should be undertaken when multiple suggestive symptoms are present simultaneously in all patients except those with cancer (7.3–8.8), or when radiology is indicative of NTM-PD (≥8.9). Symptoms of persistent sputum production, recurrent respiratory infection and haemoptysis should prompt testing for nontuberculous mycobacteria, particularly in those with underlying respiratory diseases. Symptomatic patients with bronchiectasis or previous tuberculosis/NTM-PD or those being prescribed or undergoing long-term macrolide therapy for a respiratory condition should also be tested. Testing is not warranted in patients without an underlying respiratory disorder or in those without a history of respiratory disorders unless presenting with multiple symptoms.

**Conclusions:**

Assessing patients’ risk of NTM-PD is challenging. This Delphi consensus process provides insight into symptoms and clinical characteristics that should prompt NTM-PD assessment. Timely testing and diagnosis would enable initiation of appropriate management.

## Shareable abstract

**A modified Delphi process highlighted a series of key patient groups to consider for NTM-PD testing, including those with underlying diseases, suggestive radiology and multiple symptoms, which may help to identify patients at risk** https://bit.ly/3xAW2au

## Graphical abstract

**Figure:**
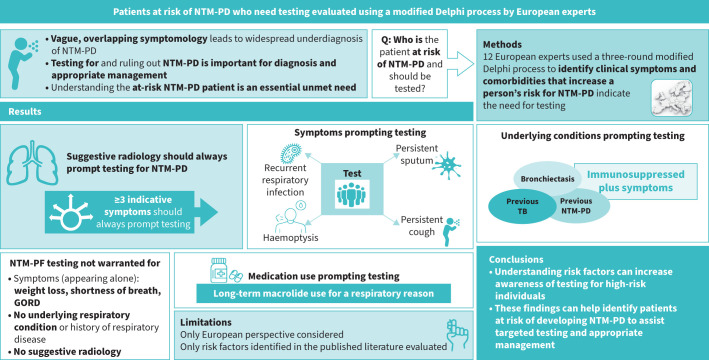
GORD: gastro-oesophageal reflux disorder; NTM-PD: nontuberculous mycobacterial pulmonary disease; Q: question; TB: tuberculosis.

## Introduction

Nontuberculous mycobacteria (NTM), of which there are more than 200 species, are ubiquitous in the environment [1]. Nontuberculous mycobacterial pulmonary disease (NTM-PD) is a serious, chronic and infectious disease defined as “rare” by the US National Organization for Rare Disorders. It is associated with substantial mortality and morbidity, as well as reduced health-related quality of life [2–4].

Incidence rates of NTM-PD are rising [5], but a low index of suspicion for infection means that the disease remains underdiagnosed [6, 7]. NTM-PD clinical symptoms are often nonspecific and overlap with other underlying respiratory conditions. Late diagnosis of NTM-PD is common, which makes effective management challenging [8, 9]. Delayed diagnosis is associated with an increase in mortality, hospitalisation and worsening patient outcomes [10, 11].

Patients previously identified as being at risk of NTM-PD include those with bronchiectasis and cystic fibrosis (CF) [12], and guidelines for these conditions encourage NTM-PD screening [13, 14]. However, neither the current COPD guidelines from 2017 [15] nor the Global Initiative for Chronic Obstructive Lung Disease (GOLD) 2023 report [16] mentioned NTM or discussed screening for NTM-PD, despite the evidence for increased risk [12].

Recent guidelines recommending the use of long-term macrolide therapy to mitigate exacerbations in chronic pulmonary diseases mandate screening and NTM testing [13, 17, 18]. However, in other chronic pulmonary diseases or high-risk groups for NTM, testing remains limited, with no comprehensive directions for NTM screening and testing in place. This expert consultation using a modified Delphi process aimed to understand the clinical factors for NTM-PD that should prompt NTM testing, the identification of which should facilitate timely and appropriate disease management.

## Methods

A European NTM-PD expert panel was enrolled to take part in a modified Delphi process to determine patient characteristics and symptoms that should prompt clinicians to test for NTM. Patients who are potentially at risk of NTM-PD were identified from the literature and explored in detail in a systematic literature review and meta-analysis (SLR-MA) (supplementary e-table 1) [19], and a survey of NTM-treating clinicians (n=455) then assessed the symptoms and patient characteristics that currently prompt NTM testing in clinical practice [20]. The SLR-MA and survey were used to develop a questionnaire to be implemented in the Delphi process, which was assisted by authors MRL and JvI [19, 20]. Three rounds of Delphi voting were conducted between July 2022 and November 2022, in line with published modified Delphi methodologies [21–23]. This was further combined with additional face-to-face and online discussions.

The questionnaires used in the Delphi process are provided in the supplementary material. Rounds 1 and 2 were developed and circulated using SurveyMonkey (SurveyMonkey Inc., San Mateo, CA, USA) after a functional pilot test by the project team at Highfield Communication. Round 3, a clarification round, was conducted over email and used a voting spreadsheet created in Microsoft Excel (Microsoft, Redmond, WA, USA). Responses were fully anonymised in all rounds by Highfield Communication before circulation of outcomes to the panel for review and discussion.

### Expert panel members

This Delphi process enrolled 12 expert panellists from across Europe. Criteria for inclusion were:

- Extensive clinical experience: each expert had >15 years of experience in NTM-PD treatment and respiratory medicine, panellists included pulmonologists, clinical microbiologists and infectious disease specialists.
- Significant input into research for NTM-PD and respiratory medicine: all panellists had participated in the development of practice guidelines for NTM-PD and/or underlying lung disease or policy and recommendation documents.
- Geographical representation: panellists were geographically distributed across Europe, including European Union and non-European Union countries.

### Voting scales and interpretation

A 9-point Likert scale was used to evaluate levels of agreement or disagreement (1=strongly disagree/testing is not required; 9=completely agree/testing should be undertaken). Voting results were aggregated into scoring tranches of 1–3, 4–6 and 7–9, as discussed by the process Chairs (MRL and JvI) and agreed by all panellists. Mean scores <4 indicated symptoms/characteristics for which NTM testing was not warranted; mean scores ≥4–<7 indicated symptoms/characteristics for which there was no clear tendency to test, with the decision depending on the specific clinical scenario (*e.g.* testing should only be carried out if other concomitant symptoms or specific clinical characteristics are present); mean scores ≥7 indicated symptoms/characteristics that experts deemed indicative of NTM-PD and would prompt them to test for NTM.

### Modified Delphi process

**FIGURE 1:**
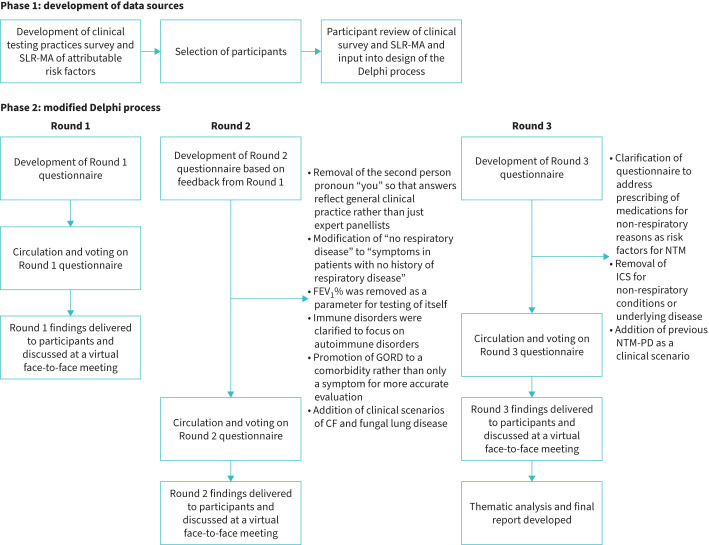
Overview of the modified Delphi process. CF: cystic fibrosis; FEV_1_: forced expiratory volume in 1 s; GORD: gastro-oesophageal reflux disorder; CF: cystic fibrosis; ICS: inhaled corticosteroids; NTM: nontuberculous mycobacteria; NTM-PD: nontuberculous mycobacterial pulmonary disease; SLR-MA: systematic literature review and meta-analysis.

An overview of the modified Delphi process used is shown in figure 1. This process was conducted in line with Conducting and REporting DElphi Studies (CREDES) guidelines [24].

### Round 1

After voting, Round 1 was discussed in a hybrid roundtable meeting. At this stage, panellists discussed modifications to the questionnaire necessary to prevent misunderstandings.

### Round 2

The questionnaire used in Round 2 was developed to expand and clarify the voting parameters and assess risk evaluation for clinicians generally (as opposed to among just the panellists; supplementary material). Other clarifications or additions made at this stage are outlined in figure 1.

Round 2 voting was then completed *via* either SurveyMonkey or, as agreed by the whole group, a Microsoft Excel spreadsheet. Participants voted with either method at their own discretion. The outcomes of Round 2 voting were then discussed in an online panel meeting.

### Round 3

A third round of voting was agreed to allow specific focus on the remaining areas of contention. These included clarifying the prescription of medications for non-respiratory reasons as risk factors for NTM-PD, the removal of a question based on inhaled corticosteroid (ICS) use in underlying diseases of non-respiratory nature (because ICS use implicitly indicates an underlying respiratory need) and the addition of a clinical scenario involving patients with previous history of NTM-PD. Panellists voted on the modified questionnaire using a Microsoft Excel spreadsheet before discussing the outcomes *via* email and in an online panel meeting. At this point in the process, there was a high level of agreement, and no further rounds of voting were deemed necessary.

## Results

**FIGURE 2:**
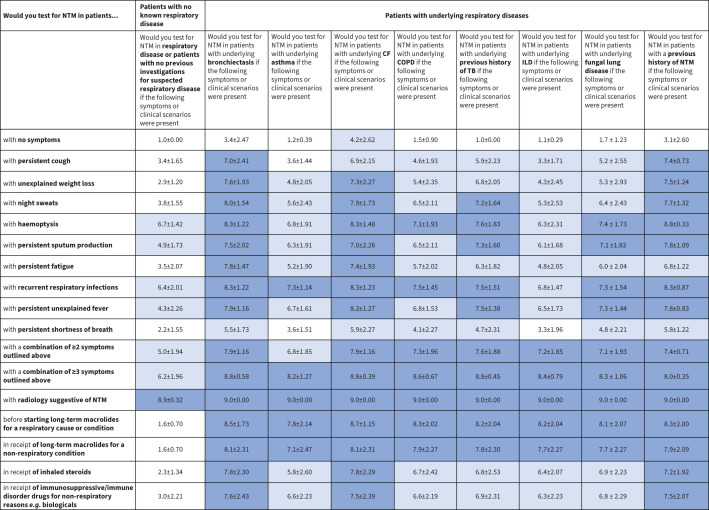
Heat map of patient characteristics and symptoms that should prompt nontuberculous mycobacteria (NTM) testing in patients with no known respiratory disease or underlying respiratory condition. Dark blue indicates a mean score ≥7, suggesting a high suspicion index to test; white indicates a mean score <4, suggesting no need for NTM testing; light blue indicates a mean score of ≥4–<7. CF: cystic fibrosis; COPD: chronic obstructive pulmonary disease; ILD: interstitial lung disease; TB: tuberculosis.

**FIGURE 3:**
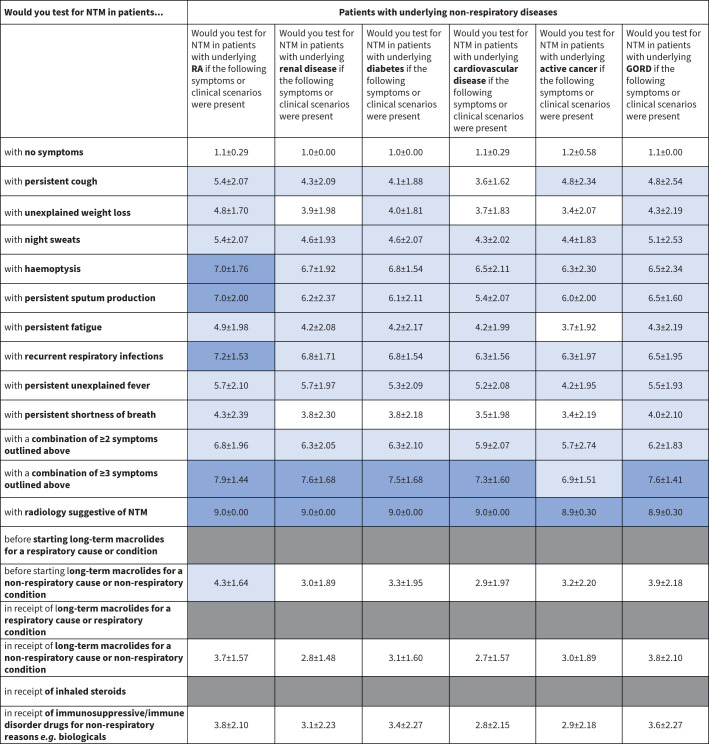
Heat map of patient characteristics and symptoms that should prompt nontuberculous mycobacteria (NTM) testing in patients with underlying conditions of a non-respiratory nature. Dark blue indicates a mean score ≥7, suggesting a high suspicion index to test; white indicates a mean score <4, suggesting no need for NTM testing; light blue indicates a mean score of ≥4–<7. GORD: gastro-oesophageal reflux disorder; RA: rheumatoid arthritis.

**FIGURE 4:**
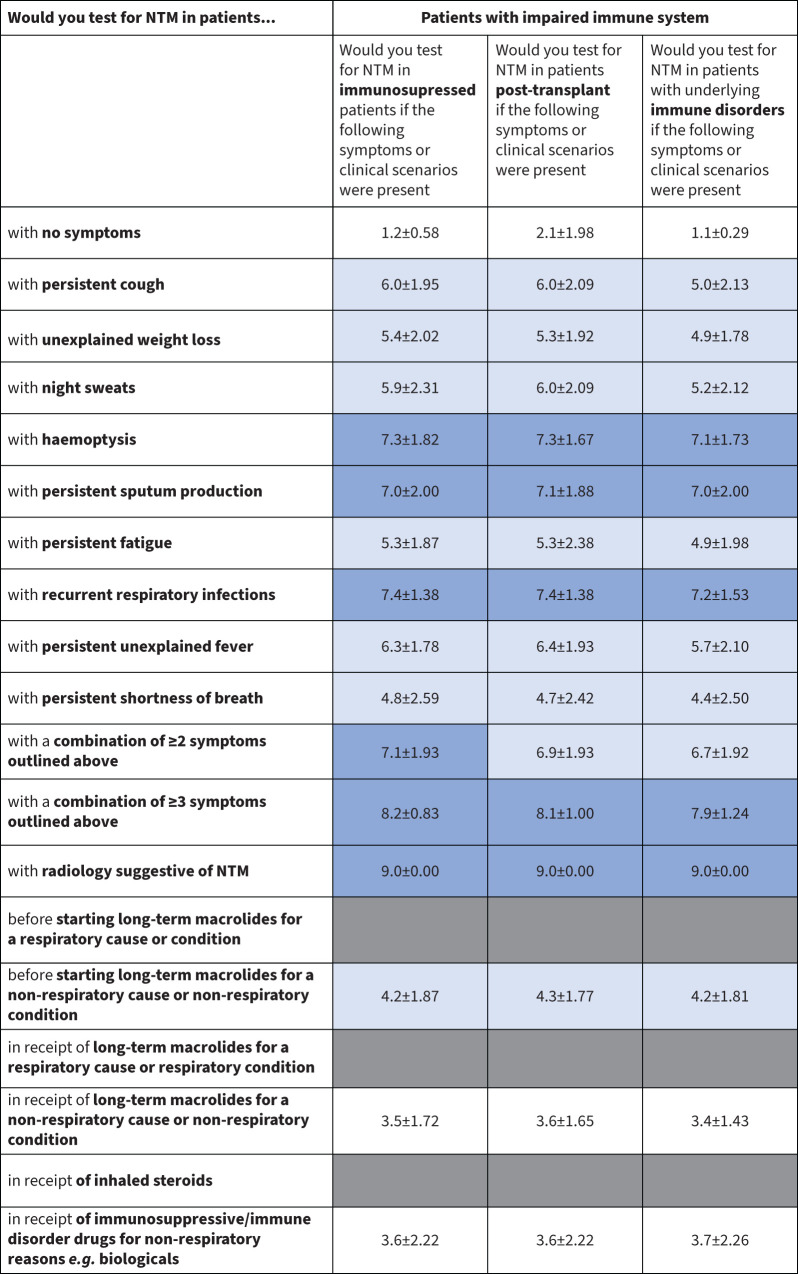
Heat map of patient characteristics and symptoms that should prompt nontuberculous mycobacteria (NTM) testing in patients with impaired immunity. Dark blue indicates a mean score ≥7, suggesting a high suspicion index to test; white indicates a mean score <4, suggesting no need for NTM testing; light blue indicates a mean score of ≥4–<7.

**FIGURE 5:**
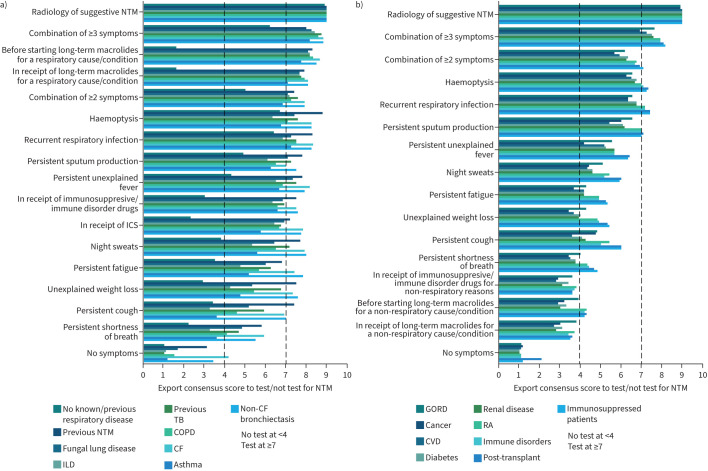
Scoring of patient characteristics and symptoms for nontuberculous mycobacterial pulmonary disease (NTM-PD) to determine the need to test in patients with underlying respiratory/non-respiratory diseases. Mean scores ≥7 suggest a high suspicion index to test for nontuberculous mycobacteria (NTM), <4 suggest no need for NTM testing and a mean score of ≥4–<7 suggests testing for NTM when overall clinical circumstances are considered. a) Scoring of patient characteristics and symptoms to test for NTM (underlying respiratory diseases). b) Scoring of patient characteristics and symptoms to test for NTM (underlying diseases of non-respiratory nature). CF: cystic fibrosis: COPD: chronic obstructive pulmonary disease; CVD: cardiovascular disease; GORD: gastro-oesophageal reflux disorder; ICS: inhaled corticosteroids; ILD: interstitial lung disease; RA: rheumatoid arthritis; TB: tuberculosis.

A heat map was developed to illustrate the panellists’ opinions on patient characteristics/symptoms that should raise suspicions of NTM-PD and prompt NTM testing (figures 2–4). These data are presented graphically for respiratory conditions (figure 5a) and non-respiratory conditions (figure 5b).

It was unanimously agreed that radiological findings suggestive of NTM-PD should prompt NTM testing through microbiological culture, regardless of attendant symptomology. The consideration of radiological abnormalities that can be present in NTM-PD include progressive or persistent infiltrates, bronchiectatic lesions, nodules, tree-in-bud lesions, mucous plugging and cavities on chest radiography or ideally on high-resolution computed tomography [7]. Experts unanimously scored this feature a 9, except in patients without underlying respiratory disease or a known history of underlying respiratory disease (mean 8.9, 95% CI 8.6–9.2), patients with cancer (mean 8.9, 95% CI 8.6–9.2) and those with gastro-oesophageal reflux disorder (GORD) (mean 8.9, 95% CI 8.6–9.2).

### Motivators to test for NTM in patients with pulmonary disease or comorbidities that are considered risk factors for NTM-PD

#### Symptoms

Across all patient types, persistent sputum production, recurrent respiratory infections and haemoptysis were symptoms that experts agreed should prompt NTM testing, even in patients with no underlying respiratory disease or history of investigative respiratory procedures (figures 2–4). In immunosuppressed patients, regardless of underlying cause, these three symptoms indicated a need for NTM testing (mean score 7.0–8.76; figures 3 and 5b). Non-respiratory conditions such as renal disease, diabetes, cardiovascular disease, cancer and GORD had lower scores (range 4–6; figures 4 and 5b). Based on the scores for this question, additional clinical assessment is needed to assess NTM-PD risk in patients presenting with these comorbidities.

Persistent cough was considered a trigger for NTM testing in patients with bronchiectasis or a previous history of NTM, but not in other patient types. By contrast, persistent shortness of breath alone was not considered to be a symptom that would prompt routine NTM testing in any patient with an underlying respiratory disorder, consistently scoring ≤5 with an overall average score of 4; it also scored similarly in patients with non-respiratory diseases. Similarly, unexplained weight loss alone was only considered a trigger for testing in patients with bronchiectasis, CF or a previous history of NTM-PD; in other patient types, symptoms such as weight loss could be attributed to other more obvious causes than NTM-PD.

#### Underlying respiratory diseases or conditions

Patients with bronchiectasis, CF, a history of NTM-PD, or tuberculosis (TB) and NTM-PD symptoms should be tested (sputum, respiratory infections and haemoptysis: mean score >7; night sweats and unexplained fever: mean score 6; figures 2 and 5a). The panel agreed that a persistent cough should not prompt NTM testing in patients with only asthma, COPD or interstitial lung disease (ILD) (range 3.3–4.6), but exacerbations or recurrent respiratory infections should prompt NTM testing in these patients and those with fungal lung disease (figures 2–4 and 3a). Haemoptysis should typically prompt testing in patients with chronic pulmonary disease (except asthma and ILD). In patients with ILD, no singular symptoms were identified to prompt testing, which should be considered when multiple symptoms or characteristic radiology findings are present (figure 2).

#### Underlying non-respiratory diseases or conditions

Without indicative radiology findings, NTM testing is not warranted in patients with a non-respiratory condition. Only those who are immunosuppressed or have an immune disorder and present with persistent sputum production, recurrent respiratory infections or haemoptysis should be tested for NTM. All other symptoms scored ≤6.9, with narrow confidence intervals indicative of strong agreement among the panellists. Testing in these patients is justified if other symptoms or clinical factors also suggest NTM-PD (figures 3 and 4).

GORD is a common comorbidity associated with conditions such as COPD [25] and may be a risk factor for NTM-PD [26]. The panellists did not consider patients with GORD a priority for NTM testing, with most scoring GORD patients between 4 and 6, unless multiple symptoms or indicative radiology findings are present.

### Prescribed medications as a motivator to test for NTM

Patients with CF or bronchiectasis using ICSs should be tested for NTM (figure 2). However, there was less agreement on whether ICS use in patients with other respiratory conditions (fungal lung disease, ILD, prior TB, asthma and COPD) should prompt NTM testing (figure 2).

Patients with underlying lung disease starting or already undergoing long-term macrolide therapy should be tested for NTM (as recommended by guidelines), even if no symptoms consistent with NTM-PD are present [13].

Experts modified the questions about patients without underlying lung disease between Round 2 and Round 3 to place greater emphasis on medication use in non-respiratory settings. Experts indicated that NTM testing in patients receiving macrolides or immunosuppressive drugs for non-respiratory conditions is not warranted, and in other patients taking immunosuppressants only those with established lung disease should be tested.

## Discussion

This Delphi process has highlighted the underlying conditions, symptoms and clinical scenarios that should prompt clinicians to test for NTM-PD. Increasing awareness of the need to test for NTM in specific patient groups relies heavily on a strong understanding of the risk factors for NTM-PD, a lack of which may result in underdiagnosis, suboptimal testing and worsening outcomes in patients with NTM-PD [17, 27].

Risk factors identified from a previously published SLR-MA were used to inform the parameters of the modified Delphi process (supplementary e-table 1) [19]. The expert panellists agreed that radiology findings indicative of NTM-PD must always prompt NTM testing, results that align with the previous survey [20] and SLR-MA [19]. Radiologists should be trained to recognise the hallmark features of NTM-PD and report them as such. Imaging, alongside microbiological analysis and identification of clinical symptoms, remains pivotal to the diagnosis, confirmation and follow-up of patients with NTM-PD [28–30]. Panellists discussed the possibility of defining combinations of multiple concurrent symptoms that should drive NTM testing, but ultimately agreed that it would be premature and potentially misleading to draw strong conclusions given the individuality of patients.

In all patients, radiological signs of NTM-PD as outlined in guidelines [7] and clinical experience should prompt testing for NTM in order to rule out infection, regardless of a lack of presenting symptoms or underlying disease.

### Symptoms prompting NTM testing

The key symptoms warranting NTM testing were persistent sputum production, recurrent respiratory infections, persistent cough and haemoptysis. Results of a clinician survey indicated that persistent cough and weight loss are important symptoms that should prompt NTM testing [20], a view largely echoed by the panellists in this Delphi process. Conversely, fatigue, which was identified in the survey as a symptom to prompt testing, was only considered an indicative symptom in bronchiectasis and CF [20]. Fatigue is a common symptom and can manifest in various other diseases [31, 32].

There was discussion among the panellists regarding whether NTM testing is appropriate in symptomatic patients in the absence of radiological evidence or in patients waiting for radiological results. In patients presenting with multiple symptoms suggestive of NTM-PD, the experts broadly agreed that testing while waiting for radiological results would be appropriate. In patients less likely (based on clinical assessment) to have NTM-PD, chest computed tomography can be performed prior to NTM testing to confirm clinical suspicion. Radiological characteristics indicative of NTM-PD, regardless of the presence of symptoms, should prompt testing. This approach aligns with data showing that patients can exhibit radiological changes without experiencing symptoms.

### Underlying respiratory disease as a motivator to test

Underlying lung disease such as bronchiectasis or previous TB or NTM-PD in symptomatic patients should prompt clinicians to test for NTM, in agreement with the previous survey and SLR-MA [19, 20]. The panellists agreed that the presence of other respiratory diseases, such as ILD (which was estimated to increase the risk of NTM-PD (OR 6.39) in the SLR-MA), should not prompt testing in all cases.

NTM testing in patients with bronchiectasis is recommended but often poorly implemented, reflecting a lack of understanding of the impact of NTM-PD [4, 33]. It was agreed that NTM testing in symptomatic patients should rule out TB relapse and/or exclude NTM (co)infection, and testing should be done in those with CF or previous NTM-PD [14]. While COPD is proposed to have a high attributable risk, the results of this Delphi process suggest that, in COPD patients, testing decisions should be made based on the presence of multiple symptoms or suggestive radiology, as opposed to the presence of COPD alone. Given the absence of guidelines for NTM testing in patients with COPD, unlike those for CF and bronchiectasis [13, 14, 34, 35], the panellists suggested that COPD guidelines should be updated to provide NTM testing guidance.

### Underlying non-respiratory comorbidities

Immunosuppressed patients with NTM-PD symptoms were the most recommended for NTM testing. Despite previous study data [19], cancer alone should not prompt NTM testing unless multiple symptoms are present.

While data indicate that GORD increases NTM-PD risk *via* aspiration [26] and older age and bronchiectasis are risk factors for NTM-PD in GORD patients [36], this study suggests GORD alone should not prompt testing.

### Medication use

#### Immunosuppressants

An association between ICS use and NTM-PD has been demonstrated in several studies [26, 37], and ICS use alone was considered a significant factor prompting NTM testing in patients with underlying lung disease. ICS use within the past year has been shown to be associated with increased risk of NTM-PD in patients with asthma, COPD and bronchiectasis [38]. Additionally, current ICS use is linked to NTM-PD in patients with obstructive lung disease [39]. It was agreed that patients using ICSs should be tested for NTM if they have underlying bronchiectasis but otherwise only in those with multiple symptoms or indicative radiology.

The use of immunosuppressive drugs for non-respiratory conditions was not considered a motivator to test for NTM, despite being considered a risk in previous work [19, 20]. This is surprising considering the high attributable NTM-PD risk associated with the use of these drugs reported by other studies [40–42], and it might imply that real-world clinical practice can sometimes be driven more by personal experience and beliefs than scientific evidence.

#### Macrolide therapy

Macrolide therapy is common in patients with underlying respiratory conditions and guidelines from 2020 suggested prescribing long-term macrolide therapy for any patient with COPD and bronchiectasis with ongoing symptoms [13, 16]. The panel scored macrolide therapy in patients with underlying respiratory conditions highly as a trigger for NTM testing. While this panel recommended NTM testing before initiating long-term macrolide therapy, which is in line with British Thoracic Society guidelines [34], real-world clinical practice often does not follow this recommendation. Recent studies found that many physicians reported testing for NTM when prescribing long-term macrolides [33, 43]. GOLD guidelines recommend the use of macrolides in patients with frequent COPD exacerbations, but do not recommend NTM testing [16]. In light of this, the expert panel proposed that COPD recommendations should be updated to include NTM testing, further emphasising the need for a greater awareness of NTM testing in high-risk groups prior to initiating macrolide therapy.

### Effective management of NTM-PD

Appropriate screening for NTM-PD can reduce inflammation-related lung damage through earlier diagnosis and treatment initiation. While the identification of patients for NTM testing remains clinically challenging, effective management of NTM-PD is important to reduce disease morbidity and mortality. Estimations of 5-year NTM-PD all-cause mortality vary: one study estimated it to be 27% [4], another reported 6% after 39 months of follow-up [44] and another demonstrated a cumulative mortality rate of 12.4%, 24.0% and 36.4% in a cohort evaluated at 5 years, 10 years and 15 years, respectively [45].

The differences between the outcomes of this panel consensus and those of the survey and SLR-MA [19, 20] reflect the drawbacks of using survey questionnaires, which are relatively blunt instruments used to gauge opinion on a single criterion alone. The consensus method used here suggests that each individual patient's clinical context is key when making decisions on NTM testing. Furthermore, the survey enrolled 455 NTM-PD-treating physicians from across Europe, the USA, Canada, Australia, New Zealand and Japan, while the SLR-MA summarised international literature published between 2011 and 2021 [19, 20]. This Delphi process included experts only from Europe, and it would be of interest to investigate whether an international panel would have differing opinions considering global epidemiological variance.

Existing bronchiectasis and CF guidelines recommend using mycobacterial culture to test for the presence of NTM in respiratory tract specimens [13, 14]. However, NTM testing can differ between clinical settings [46], and the American Thoracic Society/European Respiratory Society/European Society of Clinical Microbiology and Infectious Diseases/Infectious Diseases Society of America guidelines for NTM-PD recommend diagnosing NTM-PD based on the presence of clinical symptoms, radiological evidence and the isolation of the same organism multiple times following microbial culture of NTM isolates [29]. Hence, diagnosis of NTM-PD is a delicate evaluation that involves meeting thresholds of clinical, radiological and microbiological assessments.

### Limitations

The methodology used in this study has several limitations. First, the parameters for evaluation were drawn only from a clinical survey and SLR-MA [19, 20]. It is possible that there are other parameters indicative of NTM-PD that are not available in the public domain. Similarly, data in the public domain reporting risks may also be too limited or too poor to be identified through an SLR-MA. Second, the methodology of this Delphi process is not without bias; because it was conducted among 12 European experts, using a larger and more geographically varied panel (including experts from the Americas and the Asia-Pacific region) may have allowed more nuanced data to be collected. Finally, the methods used here did not provide comprehensive directions of how screening for NTM should be performed. The sensitivity of mycobacterial culture tests may be limited in the setting of NTM-PD depending on the sample type.

In summary, experts identified key characteristics with high levels of agreement to prompt testing, including radiological changes consistent with NTM-PD, the presence of three or more symptoms consistent with NTM-PD, bronchiectasis, previous TB or previous NTM-PD, patients receiving or being prescribed long-term macrolides for a respiratory purpose and those presenting with symptoms such as haemoptysis and recurrent respiratory infections.

### Conclusion

The results of this Delphi process provide additional guidance to healthcare professionals who manage patients at risk of NTM-PD, especially in primary and secondary care settings where timely NTM testing is most needed. Radiology suggestive of NTM-PD must always prompt NTM testing. Patients presenting with haemoptysis, persistent sputum production, recurrent respiratory infections and/or a combination of symptoms consistent with NTM-PD should be tested. Those with underlying respiratory conditions who are prescribed or currently receiving macrolide therapy should also be tested. The consensus reached will hopefully assist physicians by providing suggestions that can be readily implemented in clinical practice. Improved clinical awareness regarding which NTM-PD-associated symptoms and comorbidities should prompt testing can guide earlier diagnosis, more effective management and better patient outcomes.

## Supplementary material

10.1183/23120541.00791-2023.Supp1 **Please note:** supplementary material is not edited by the Editorial Office, and is uploaded as it has been supplied by the author.
Supplementary material 00791-2023.SUPPLEMENT
