# “Patients at risk of nontuberculous mycobacterial pulmonary disease who need testing evaluated using a modified Delphi process by European experts.” Michael R. Loebinger, Stefano Aliberti, Charles Haworth, Mateja Jankovic Makek, Christoph Lange, Natalie Lorent, Apostolos Papavasileiou, Eva Polverino, Gernot Rohde, Nicolas Veziris, Dirk Wagner and Jakko van Ingen. *ERJ Open Res* 2024; 10: 00791-2023.

**DOI:** 10.1183/23120541.50791-2023

**Published:** 2025-01-20

## Abstract

**Figure:**
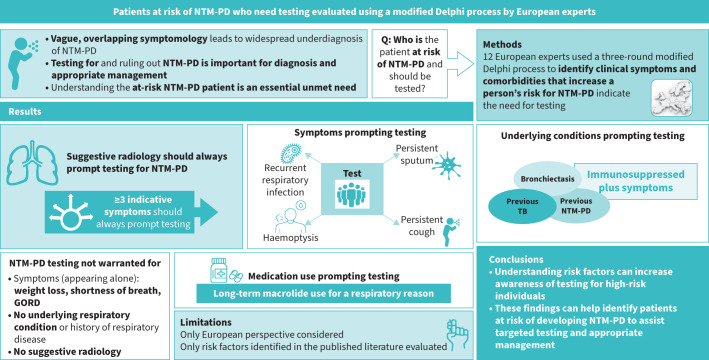
GORD: gastro-oesophageal reflux disorder; NTM-PD: nontuberculous mycobacterial pulmonary disease; Q: question; TB: tuberculosis.

GORD: gastro-oesophageal reflux disorder; NTM-PD: nontuberculous mycobacterial pulmonary disease; Q: question; TB: tuberculosis.

This article was originally published with an error in the graphical abstract in which one instance of “NTM-PD” had been incorrectly changed to “NTM-PF”. We apologise for this error. The corrected graphical abstract is shown and has been corrected in the article itself.

