# Supplementary material for: Patients at risk of nontuberculous mycobacterial pulmonary disease who need testing evaluated using a modified Delphi process by European experts
Source: ERJ Open Res. 2024 Sep 23;10(5):00791-2023. doi: 10.1183/23120541.00791-2023 (PMC11417603; doi:10.1183/23120541.00791-2023)
Supplement: Supplementary file 1 [file 00791-2023.SUPPLEMENT.pdf]

SUPPLEMENTARY INFORMATION

Patients at risk of NTM-PD who need testing evaluated using a modified Delphi process by European experts

Michael R Loebinger,[1] Stefano Aliberti,[2,3] Charles Haworth,[4,5] Mateja Jankovic Makek,[6,7] Christoph Lange,[8,9,10,11] Natalie Lorent,[12,13] Apostolos Papavasileiou,[14] Eva Polverino,[15] Gernot Rohde,[16] Nicolas Veziris,[17] Dirk Wagner,[18] Jakko van Ingen.[19]

Affiliations

1. Royal Brompton Hospital and NHLI, Imperial College London, London, UK
2. Department of Biomedical Sciences, Humanitas University, Via Rita Levi Montalcini 4, 20072 Pieve Emanuele, Milan, Italy
3. IRCCS Humanitas Research Hospital, Respiratory Unit, Via Manzoni 56, 20089 Rozzano, Milan, Italy.
4. Cambridge Centre for Lung Infection, Royal Papworth Hospital, Cambridge, UK.
5. Department of Medicine, University of Cambridge, Cambridge, UK.
6. Clinic for Respiratory Diseases, University Hospital Center Zagreb, Zagreb, Croatia.
7. University of Zagreb, School of Medicine, Zagreb, Croatia
8. Division of Clinical Infectious Diseases, Research Center Borstel, Borstel, Germany.
9. German Center for Infection Research (DZIF).
10. Respiratory Medicine and International Health, University of Lübeck, Lübeck, Germany.
11. Global TB Program, Baylor College of Medicine and Texas Children's Hospital, Houston, TX, USA.
12. Department of Respiratory Medicine, University Hospitals Leuven, Leuven, Belgium.
13. Faculty of Medicine, Katholieke Universiteit Leuven (KUL), Leuven, Belgium
14. Department of Mycobacterial Infections, Sotiria Athens Hospital of Chest Diseases, Athens, Greece.
15. Pneumology Dept, Hospital Universitari Vall d'Hebron, Vall d'Hebron Institut de Recerca (VHIR), Vall d'Hebron Barcelona Hospital Campus, Ciber de Enfermedades Respiratorias CIBERES, Barcelona, Spain.
16. Goethe University Frankfurt, University Hospital, Department of Respiratory Medicine, Frankfurt am Main, Germany.
17. Sorbonne Université, Centre d'Immunologie et des Maladies Infectieuses (Cimi-Paris), UMR 1135, Département de Bactériologie, Hôpital Saint-Antoine, Centre National de Référence des Mycobactéries, APHP.Sorbonne Université, Paris, France)
18. Division of Infectious Diseases, Department of Internal Medicine II, Freiburg University Medical Centre, Freiburg, Germany.
19. Department of Medical Microbiology, Radboud University Medical Center, Nijmegen, the Netherlands

Correspondence to: Michael R. Loebinger, MA, FRCP, PhD; Royal Brompton Hospital, Sydney St, London SW3 6NP; email: m.loebinger@rbht.nhs.uk

Contents

eTable 1. Meta-analyses of Identified Attributable Risk Factors: Overview of Outcomes 3

Delphi Process Round 1 questionnaire 5

Delphi Process Round 2 questionnaire 93

Delphi Process Round 3 voting sheet 146

e-Table 1 Meta-analyses of Identified Attributable Risk Factors: Overview of Outcomes

Identified Risk Factor	Baseline Population	Combined OR	95% CI	I^2 (%)
Non-cystic fibrosis bronchiectasis	**General population; symptoms of TB**	**21.43**	**5.90-77.82**	**95**
History of TB	**General population; symptoms of TB; rheumatoid arthritis; COPD**	**12.69**	**2.39-67.26**	**99**
Interstitial lung disease	**General population; rheumatoid arthritis; COPD**	**6.39**	**2.65-15.37**	**97**
COPD	**General population; symptoms of TB; rheumatoid arthritis**	**6.63**	**4.57-9.63**	**96**
Pneumonia	**General population; COPD; CF**	**5.54**	**2.72-11.26**	**95**
Solid tumors	**General population; COPD**	**4.66**	**1.04-20.94**	**89**
Inhaled corticosteroids	**General population; pulmonary disease**	**4.46**	**2.13-9.35**	**97**
Asthma	**General population; symptoms of TB**	**4.53**	**2.24-9.15**	**98**
Solid organ transplant	Lung transplant; solid organ transplant	3.69	0.31-44.12	92
Oral corticosteroids	General population; rheumatoid arthritis	3.37	0.82-13.75	96
Low body mass index (<18 kg/m^2)	**General population; COPD**	**3.04**	**1.95-4.73**	**88**
Infection with *Staphylococcus aureus*	CF	2.42	0.55-10.63	84
Immunosuppression (others)	General population; SOTRs	2.60	0.69-9.79	90
Immune disorders	General population; TB	2.36	0.98-5.64	84
Use of anti-tumor necrosis factor-α treatment	**Rheumatoid arthritis**	**2.13**	**1.24-3.65**	**0**
Radiological findings	TB	2.05	0.92-4.59	66
Cardiovascular disease	**Rheumatoid arthritis; general population**	**1.73**	**1.01-2.97**	**50**
Sex (female)	**Non-cystic fibrosis bronchiectasis; CF; solid organ transplant; TB**	**1.70**	**1.01-2.86**	**87**
Renal disease	General population; rheumatoid arthritis; transplant; TB	1.62	0.87-3.03	82
Cancer (all)	General population; solid organ transplant; TB; CF	1.37	0.70-2.68	85

Sex (overall)	Non-cystic fibrosis bronchiectasis; CF; TB; solid organ transplant	1.28	0.99-1.66	78
Infection with *Pseudomonas aeruginosa*	General population; COPD; CF	1.12	0.87-1.44	95
Sex (male)	CF; TB	1.10	0.95-1.28	0
Diabetes	General population; rheumatoid arthritis; solid organ transplant; TB	1.04	0.63-1.72	88
Forced expiratory volume in 1 second (FEV_1)% of predicted	CF	1.01	0.97-1.05	97
Higher body mass index (>24 kg/m^2)	**General population; non-cystic fibrosis bronchiectasis**	**0.73**	**0.58-0.97**	**91**
Macrolide use	CF	0.80	0.47-1.39	96

Bold factors are statistically significant ($P < 0.05$).

CF = cystic fibrosis; CI = confidence interval; COPD = chronic obstructive pulmonary disease; NTM-PD = non-tuberculous mycobacterial pulmonary disease; OR = odds ratio; SOTRs = solid organ transplant recipients; TB = tuberculosis.

Delphi process – Round 1 Questionnaire

Section 1: NTM symptoms in possible at-risk patients

In this section questions explore symptoms for NTM-PD that were identified from the survey and the meta-analysis and explore when you might consider testing in patients with underlying comorbidities e.g. respiratory disease, other diseases, reductions in effective bodily functions as well as those in receipt of specific medications.

* 1. In order to facilitate discussion within the Delphi panel meetings, please add your name below. (Your identity is only visible to Highfield to facilitate effective development of the meeting agenda and discussion topics)

NO BACKGROUND RESPIRATORY COMORBIDITY

In patients with no background respiratory comorbidities which symptoms would prompt you to test for NTM?

* 2. No symptoms

Strongly disagree 1	2	3	4	5	6	7	8	Strongly Agree 9
★	★	★	★	★	★	★	★	★

* 3. Persistent cough

Strongly Disagree 1	2	3	4	5	6	7	8	Strongly Agree 9
★	★	★	★	★	★	★	★	★

* 4. Weight loss for no clear reason

Strongly Disagree 1	2	3	4	5	6	7	8	Strongly Agree 9
★	★	★	★	★	★	★	★	★

* 5. Night sweats

Strongly Disagree 1	2	3	4	5	6	7	8	Strongly Agree 9
★	★	★	★	★	★	★	★	★

* 6. Haemoptysis

Strongly Disagree 1	2	3	4	5	6	7	8	Strongly Agree 9
★	★	★	★	★	★	★	★	★

* 7. Increased or purulent sputum

Strongly Disagree 1	2	3	4	5	6	7	8	Strongly Agree 9
★	★	★	★	★	★	★	★	★

* 8. Persistent fatigue

Strongly Disagree 1	2	3	4	5	6	7	8	Strongly Agree 9
★	★	★	★	★	★	★	★	★

* 9. Increased frequency of exacerbations

Strongly Disagree 1	2	3	4	5	6	7	8	Strongly Agree 9
★	★	★	★	★	★	★	★	★

* 10. Fever

Strongly Disagree 1	2	3	4	5	6	7	8	Strongly Agree 9
★	★	★	★	★	★	★	★	★

* 11. Hoarseness

Strongly Disagree 1	2	3	4	5	6	7	8	Strongly Agree 9
★	★	★	★	★	★	★	★	★

* 12. GERD

Strongly Disagree 1	2	3	4	5	6	7	8	Strongly Agree 9
★	★	★	★	★	★	★	★	★

* 13. Decline in FEV1 of >20%

Strongly Disagree 1	2	3	4	5	6	7	8	Strongly Agree 9
★	★	★	★	★	★	★	★	★

* 14. Shortness of breath

Strongly Disagree 1	2	3	4	5	6	7	8	Strongly Agree 9
★	★	★	★	★	★	★	★	★

* 15. Combination of ≥2 or more symptoms outlined above

Strongly Disagree 1	2	3	4	5	6	7	8	Strongly Agree 9
☆	☆	☆	☆	☆	☆	☆	☆	☆

* 16. Radiology suggestive of NTM

Strongly Disagree 1	2	3	4	5	6	7	8	Strongly Agree 9
☆	☆	☆	☆	☆	☆	☆	☆	☆

NO BACKGROUND RESPIRATORY COMORBIDITY AND MEDICATIONS

Which of the following medications patients with no background respiratory comorbidities are taking would make you consider testing for NTM?

Patients in receipt of...

* 17. Macrolide therapy

Strongly Disagree 1	2	3	4	5	6	7	8	Strongly Agree 9
☆	☆	☆	☆	☆	☆	☆	☆	☆

* 18. Any additional considerations? E.g. dose, type of macrolide, duration of use...

* 19. Inhaled corticosteroids

Strongly Disagree 1	2	3	4	5	6	7	8	Strongly Agree 9
☆	☆	☆	☆	☆	☆	☆	☆	☆

* 20. Any additional considerations? E.g. dose, duration of use, specific steroids?

* 21. Oral corticosteroids

Strongly Disagree 1	2	3	4	5	6	7	8	Strongly Agree 9
☆	☆	☆	☆	☆	☆	☆	☆	☆

* 22. Any additional considerations? E.g. dose, duration of use, specific steroids?

* 23. Monoclonal antibodies

Strongly Disagree 1	2	3	4	5	6	7	8	Strongly Agree 9
☆	☆	☆	☆	☆	☆	☆	☆	☆

* 24. Any additional considerations? E.g. dose, duration of use, specific medications over others?

* 25. Biologic therapy e.g. immunomodulators

Strongly Disagree 1	2	3	4	5	6	7	8	Strongly Agree 9
☆	☆	☆	☆	☆	☆	☆	☆	☆

* 26. Any additional considerations? E.g. dose, duration of use, specific medications over others?

* 27. IV corticosteroids

Strongly Disagree 1	2	3	4	5	6	7	8	Strongly Agree 9
☆	☆	☆	☆	☆	☆	☆	☆	☆

* 28. Any additional considerations? E.g. dose, duration of use, specific steroids?

* 29. Immunosuppressant therapy

Strongly Disagree 1	2	3	4	5	6	7	8	Strongly Agree 9
☆	☆	☆	☆	☆	☆	☆	☆	☆

* 30. Any additional considerations? E.g. dose, duration of use, specific medications over others?

* 31. Other medications not indicated above – please list

NON-CF BRONCHIECTASIS (NCFBE)

In patients with NCFBE which symptoms would prompt you to test for NTM?

* 32. No symptoms

Strongly Disagree 1	2	3	4	5	6	7	8	Strongly Agree 9
★	★	★	★	★	★	★	★	★

* 33. Persistent cough

Strongly Disagree 1	2	3	4	5	6	7	8	Strongly Agree 9
★	★	★	★	★	★	★	★	★

* 34. Weight loss for no clear reason

Strongly Disagree 1	2	3	4	5	6	7	8	Strongly Agree 9
★	★	★	★	★	★	★	★	★

* 35. Night sweats

Strongly Disagree 1	2	3	4	5	6	7	8	Strongly Agree 9
★	★	★	★	★	★	★	★	★

* 36. Haemoptysis

Strongly Disagree 1	2	3	4	5	6	7	8	Strongly Agree 9
★	★	★	★	★	★	★	★	★

* 37. Increased or purulent sputum

Strongly Disagree 1	2	3	4	5	6	7	8	Strongly Agree 9
★	★	★	★	★	★	★	★	★

* 38. Persistent fatigue

Strongly Disagree 1	2	3	4	5	6	7	8	Strongly Agree 9
☆	☆	☆	☆	☆	☆	☆	☆	☆

* 39. Increased frequency of exacerbations

Strongly Disagree 1	2	3	4	5	6	7	8	Strongly Agree 9
☆	☆	☆	☆	☆	☆	☆	☆	☆

* 40. Fever

Strongly Disagree 1	2	3	4	5	6	7	8	Strongly Agree 9
☆	☆	☆	☆	☆	☆	☆	☆	☆

* 41. Hoarseness

Strongly Disagree 1	2	3	4	5	6	7	8	Strongly Agree 9
☆	☆	☆	☆	☆	☆	☆	☆	☆

* 42. GERD

Strongly Disagree 1	2	3	4	5	6	7	8	Strongly Agree 9
☆	☆	☆	☆	☆	☆	☆	☆	☆

* 43. Decline in FEV1 of >20%

Strongly Disagree 1	2	3	4	5	6	7	8	Strongly Agree 9
☆	☆	☆	☆	☆	☆	☆	☆	☆

* 44. Shortness of breath

Strongly Disagree 1	2	3	4	5	6	7	8	Strongly Agree 9
☆	☆	☆	☆	☆	☆	☆	☆	☆

* 45. Combination of ≥2 or more symptoms outlined above

Strongly Disagree 1	2	3	4	5	6	7	8	Strongly Agree 9
★	★	★	★	★	★	★	★	★

* 46. Radiology suggestive of NTM

Strongly Disagree 1	2	3	4	5	6	7	8	Strongly Agree 9
★	★	★	★	★	★	★	★	★

NON-CF BRONCHIECTASIS (NCFBE) AND MEDICATIONS

Which of the following medications patients with NCFBE are taking would make you consider testing for NTM?

Patients in receipt of...

* 47. Macrolide therapy

Strongly Disagree 1	2	3	4	5	6	7	8	Strongly Agree 9
★	★	★	★	★	★	★	★	★

* 48. Any additional considerations? E.g. dose, type of macrolide, duration of use...

* 49. Inhaled corticosteroids

Strongly Disagree 1	2	3	4	5	6	7	8	Strongly Agree 9
★	★	★	★	★	★	★	★	★

* 50. Any additional considerations? E.g. dose, duration of use, specific steroids?

* 51. Oral corticosteroids

Strongly Disagree 1	2	3	4	5	6	7	8	Strongly Agree 9
★	★	★	★	★	★	★	★	★

* 52. Any additional considerations? E.g. dose, duration of use, specific steroids?

* 53. Monoclonal antibodies

Strongly Disagree 1	2	3	4	5	6	7	8	Strongly Agree 9
★	★	★	★	★	★	★	★	★

* 54. Any additional considerations? E.g. dose, duration of use, specific medications over others?

* 55. Biologic therapy e.g. immunomodulators

Strongly Disagree 1	2	3	4	5	6	7	8	Strongly Agree 9
★	★	★	★	★	★	★	★	★

* 56. Any additional considerations? E.g. dose, duration of use, specific medications over others?

* 57. IV corticosteroids

Strongly Disagree 1	2	3	4	5	6	7	8	Strongly Agree 9
★	★	★	★	★	★	★	★	★

* 58. Any additional considerations? E.g. dose, duration of use, specific steroids?

* 59. Immunosuppressant therapy

Strongly Disagree 1	2	3	4	5	6	7	8	Strongly Agree 9
★	★	★	★	★	★	★	★	★

* 60. Any additional considerations? E.g. dose, duration of use, specific medications over others?

* 61. Other medications not indicated above – please list

ASTHMA

In patients with Asthma which symptoms would prompt you to test for NTM?

* 62. No symptoms

Strongly Disagree 1	2	3	4	5	6	7	8	Strongly Agree 9
★	★	★	★	★	★	★	★	★

* 63. Persistent cough

Strongly Disagree 1	2	3	4	5	6	7	8	Strongly Agree 9
★	★	★	★	★	★	★	★	★

* 64. Weight loss for no clear reason

Strongly Disagree 1	2	3	4	5	6	7	8	Strongly Agree 9
★	★	★	★	★	★	★	★	★

* 65. Night sweats

Strongly Disagree 1	2	3	4	5	6	7	8	Strongly Agree 9
★	★	★	★	★	★	★	★	★

* 66. Haemoptysis

Strongly Disagree 1	2	3	4	5	6	7	8	Strongly Agree 9
★	★	★	★	★	★	★	★	★

* 67. Increased or purulent sputum

Strongly Disagree 1	2	3	4	5	6	7	8	Strongly Agree 9
★	★	★	★	★	★	★	★	★

* 68. Persistent fatigue

Strongly Disagree 1	2	3	4	5	6	7	8	Strongly Agree 9
☆	☆	☆	☆	☆	☆	☆	☆	☆

* 69. Increased frequency of exacerbations

Strongly Disagree 1	2	3	4	5	6	7	8	Strongly Agree 9
☆	☆	☆	☆	☆	☆	☆	☆	☆

* 70. Fever

Strongly Disagree 1	2	3	4	5	6	7	8	Strongly Agree 9
☆	☆	☆	☆	☆	☆	☆	☆	☆

* 71. Hoarseness

Strongly Disagree 1	2	3	4	5	6	7	8	Strongly Agree 9
☆	☆	☆	☆	☆	☆	☆	☆	☆

* 72. GERD

Strongly Disagree 1	2	3	4	5	6	7	8	Strongly Agree 9
☆	☆	☆	☆	☆	☆	☆	☆	☆

* 73. Decline in FEV1 of >20%

Strongly Disagree 1	2	3	4	5	6	7	8	Strongly Agree 9
☆	☆	☆	☆	☆	☆	☆	☆	☆

* 74. Shortness of breath

Strongly Disagree 1	2	3	4	5	6	7	8	Strongly Agree 9
☆	☆	☆	☆	☆	☆	☆	☆	☆

* 75. Combination of ≥2 or more symptoms outlined above

Strongly Disagree 1	2	3	4	5	6	7	8	Strongly Agree 9
★	★	★	★	★	★	★	★	★

* 76. Radiology suggestive of NTM

Strongly Disagree 1	2	3	4	5	6	7	8	Strongly Agree 9
★	★	★	★	★	★	★	★	★

ASTHMA AND MEDICATIONS

Which of the following medications patients with asthma are taking would make you consider testing for NTM?

Patients in receipt of…

* 77. Macrolide therapy

Strongly Disagree 1	2	3	4	5	6	7	8	Strongly Agree 9
☆	☆	☆	☆	☆	☆	☆	☆	☆

* 78. Any additional considerations? E.g. dose, type of macrolide, duration of use…

* 79. Inhaled corticosteroids

Strongly Disagree 1	2	3	4	5	6	7	8	Strongly Agree 9
☆	☆	☆	☆	☆	☆	☆	☆	☆

* 80. Any additional considerations? E.g. dose, duration of use, specific steroids?

* 81. Oral corticosteroids

Strongly Disagree 1	2	3	4	5	6	7	8	Strongly Agree 9
☆	☆	☆	☆	☆	☆	☆	☆	☆

* 82. Any additional considerations? E.g. dose, duration of use, specific steroids?

* 83. Monoclonal antibodies

Strongly Disagree 1	2	3	4	5	6	7	8	Strongly Agree 9
☆	☆	☆	☆	☆	☆	☆	☆	☆

* 84. Any additional considerations? E.g. dose, duration of use, specific medications over others?

* 85. Biologic therapy e.g. immunomodulators

Strongly Disagree 1	2	3	4	5	6	7	8	Strongly Agree 9
★	★	★	★	★	★	★	★	★

* 86. Any additional considerations? E.g. dose, duration of use, specific medications over others?

* 87. IV corticosteroids

Strongly Disagree 1	2	3	4	5	6	7	8	Strongly Agree 9
★	★	★	★	★	★	★	★	★

* 88. Any additional considerations? E.g. dose, duration of use, specific steroids?

* 89. Immunosuppressant therapy

Strongly Disagree 1	2	3	4	5	6	7	8	Strongly Agree 9
★	★	★	★	★	★	★	★	★

* 90. Any additional considerations? E.g. dose, duration of use, specific medications over others?

* 91. Other medications not indicated above – please list

COPD

In patients with COPD which symptoms would prompt you to test for NTM?

* 92. No Symptoms

Strongly Disagree 1	2	3	4	5	6	7	8	Strongly Agree 9
★	★	★	★	★	★	★	★	★

* 93. Persistent cough

Strongly Disagree 1	2	3	4	5	6	7	8	Strongly Agree 9
★	★	★	★	★	★	★	★	★

* 94. Weight loss for no clear reason

Strongly Disagree 1	2	3	4	5	6	7	8	Strongly Agree 9
★	★	★	★	★	★	★	★	★

* 95. Night sweats

Strongly Disagree 1	2	3	4	5	6	7	8	Strongly Agree 9
★	★	★	★	★	★	★	★	★

* 96. Haemoptysis

Strongly Disagree 1	2	3	4	5	6	7	8	Strongly Agree 9
★	★	★	★	★	★	★	★	★

* 97. Increased or purulent sputum

Strongly Disagree 1	2	3	4	5	6	7	8	Strongly Agree 9
★	★	★	★	★	★	★	★	★

* 98. Persistent fatigue

Strongly Disagree 1	2	3	4	5	6	7	8	Strongly Agree 9
☆	☆	☆	☆	☆	☆	☆	☆	☆

* 99. Increased frequency of exacerbations

Strongly Disagree 1	2	3	4	5	6	7	8	Strongly Agree 9
☆	☆	☆	☆	☆	☆	☆	☆	☆

* 100. Fever

Strongly Disagree 1	2	3	4	5	6	7	8	Strongly Agree 9
☆	☆	☆	☆	☆	☆	☆	☆	☆

* 101. Hoarseness

Strongly Disagree 1	2	3	4	5	6	7	8	Strongly Agree 9
☆	☆	☆	☆	☆	☆	☆	☆	☆

* 102. GERD

Strongly Disagree 1	2	3	4	5	6	7	8	Strongly Agree 9
☆	☆	☆	☆	☆	☆	☆	☆	☆

* 103. Decline in FEV1 of >20%

Strongly Disagree 1	2	3	4	5	6	7	8	Strongly Agree 9
☆	☆	☆	☆	☆	☆	☆	☆	☆

* 104. Shortness of breath

Strongly Disagree 1	2	3	4	5	6	7	8	Strongly Agree 9
☆	☆	☆	☆	☆	☆	☆	☆	☆

* 105. Combination of ≥2 or more symptoms outlined above

Strongly Disagree 1	2	3	4	5	6	7	8	Strongly Agree 9
★	★	★	★	★	★	★	★	★

* 106. Radiology suggestive of NTM

Strongly Disagree 1	2	3	4	5	6	7	8	Strongly Agree 9
★	★	★	★	★	★	★	★	★

COPD AND MEDICATIONS

Which of the following medications patients with COPD are taking would make you consider testing for NTM?

Patients in receipt of...

* 107. Macrolide therapy

Strongly Disagree 1	2	3	4	5	6	7	8	Strongly Agree 9
☆	☆	☆	☆	☆	☆	☆	☆	☆

* 108. Any additional considerations? E.g. dose, type of macrolide, duration of use...

* 109. Inhaled corticosteroids

Strongly Disagree 1	2	3	4	5	6	7	8	Strongly Agree 9
☆	☆	☆	☆	☆	☆	☆	☆	☆

* 110. Any additional considerations? E.g. dose, duration of use, specific steroids?

* 111. Oral corticosteroids

Strongly Disagree 1	2	3	4	5	6	7	8	Strongly Agree 9
☆	☆	☆	☆	☆	☆	☆	☆	☆

* 112. Any additional considerations? E.g. dose, duration of use, specific steroids?

* 113. Monoclonal antibodies

Strongly Disagree 1	2	3	4	5	6	7	8	Strongly Agree 9
☆	☆	☆	☆	☆	☆	☆	☆	☆

* 114. Any additional considerations? E.g. dose, duration of use, specific medications over others?

* 115. Biologic therapy e.g. immunomodulators

Strongly Disagree 1	2	3	4	5	6	7	8	Strongly Agree 9
★	★	★	★	★	★	★	★	★

* 116. Any additional considerations? E.g. dose, duration of use, specific medications over others?

* 117. IV corticosteroids

Strongly Disagree 1	2	3	4	5	6	7	8	Strongly Agree 9
★	★	★	★	★	★	★	★	★

* 118. Any additional considerations? E.g. dose, duration of use, specific steroids?

* 119. Immunosuppressant therapy

Strongly Disagree 1	2	3	4	5	6	7	8	Strongly Agree 9
★	★	★	★	★	★	★	★	★

* 120. Any additional considerations? E.g. dose, duration of use, specific medications over others?

* 121. Other medications not indicated above – please list

PREVIOUS HISTORY OF TB

In patients with a previous history of TB which symptoms would prompt you to test for NTM?

* 122. No symptoms

Strongly Disagree 1	2	3	4	5	6	7	8	Strongly Agree 9
★	★	★	★	★	★	★	★	★

* 123. Persistent cough

Strongly Disagree 1	2	3	4	5	6	7	8	Strongly Agree 9
★	★	★	★	★	★	★	★	★

* 124. Weight loss for no clear reason

Strongly Disagree 1	2	3	4	5	6	7	8	Strongly Agree 9
★	★	★	★	★	★	★	★	★

* 125. Night sweats

Strongly Disagree 1	2	3	4	5	6	7	8	Strongly Agree 9
★	★	★	★	★	★	★	★	★

* 126. Haemoptysis

Strongly Disagree 1	2	3	4	5	6	7	8	Strongly Agree 9
★	★	★	★	★	★	★	★	★

* 127. Increased or purulent sputum

Strongly Disagree 1	2	3	4	5	6	7	8	Strongly Agree 9
★	★	★	★	★	★	★	★	★

* 128. Persistent fatigue

Strongly Disagree 1	2	3	4	5	6	7	8	Strongly Agree 9

* 129. Increased frequency of exacerbations

Strongly Disagree 1	2	3	4	5	6	7	8	Strongly Agree 9

* 130. Fever

Strongly Disagree 1	2	3	4	5	6	7	8	Strongly Agree 9

* 131. Hoarseness

Strongly Disagree 1	2	3	4	5	6	7	8	Strongly Agree 9

* 132. GERD

Strongly Disagree 1	2	3	4	5	6	7	8	Strongly Agree 9

* 133. Decline in FEV1 of >20%

Strongly Disagree 1	2	3	4	5	6	7	8	Strongly Agree 9

* 134. Shortness of breath

Strongly Disagree 1	2	3	4	5	6	7	8	Strongly Agree 9

* 135. Combination of ≥2 or more symptoms outlined above

Strongly Disagree 1	2	3	4	5	6	7	8	Strongly Agree 9
☆	☆	☆	☆	☆	☆	☆	☆	☆

* 136. Radiology suggestive of NTM

Strongly Disagree 1	2	3	4	5	6	7	8	Strongly Agree 9
☆	☆	☆	☆	☆	☆	☆	☆	☆

PREVIOUS HISTORY OF TB AND MEDICATIONS

Which of the following medications patients with a previous history of TB are taking would make you consider testing for NTM?

* 137. Macrolide therapy

Strongly Disagree 1	2	3	4	5	6	7	8	Strongly Agree 9
★	★	★	★	★	★	★	★	★

* 138. Any additional considerations? E.g. dose, type of macrolide, duration of use…

* 139. Inhaled corticosteroids

Strongly Disagree 1	2	3	4	5	6	7	8	Strongly Agree 9
★	★	★	★	★	★	★	★	★

* 140. Any additional considerations? E.g. dose, duration of use, specific steroids?

* 141. Oral corticosteroids

Strongly Disagree 1	2	3	4	5	6	7	8	Strongly Agree 9
★	★	★	★	★	★	★	★	★

* 142. Any additional considerations? E.g. dose, duration of use, specific steroids?

* 143. Monoclonal antibodies

Strongly Disagree 1	2	3	4	5	6	7	8	Strongly Agree 9
★	★	★	★	★	★	★	★	★

* 144. Any additional considerations? E.g. dose, duration of use, specific medications over others?

* 145. Biologic therapy e.g. immunomodulators

Strongly Disagree 1	2	3	4	5	6	7	8	Strongly Agree 9
☆	☆	☆	☆	☆	☆	☆	☆	☆

* 146. Any additional considerations? E.g. dose, duration of use, specific medications over others?

* 147. IV corticosteroids

Strongly Disagree 1	2	3	4	5	6	7	8	Strongly Agree 9
☆	☆	☆	☆	☆	☆	☆	☆	☆

* 148. Any additional considerations? E.g. dose, duration of use, specific steroids?

* 149. Immunosuppressant therapy

Strongly Disagree 1	2	3	4	5	6	7	8	Strongly Agree 9
☆	☆	☆	☆	☆	☆	☆	☆	☆

* 150. Any additional considerations? E.g. dose, duration of use, specific medications over others?

* 151. Other medications not indicated above – please list

IMMUNOSUPPRESSED PATIENTS

In immunosuppressed patients which symptoms would prompt you to test for NTM?

* 152. No symptoms

Strongly Disagree 1	2	3	4	5	6	7	8	Strongly Agree 9
★	★	★	★	★	★	★	★	★

* 153. Persistent cough

Strongly Disagree 1	2	3	4	5	6	7	8	Strongly Agree 9
★	★	★	★	★	★	★	★	★

* 154. Weight loss for no clear reason

Strongly Disagree 1	2	3	4	5	6	7	8	Strongly Agree 9
★	★	★	★	★	★	★	★	★

* 155. Night sweats

Strongly Disagree 1	2	3	4	5	6	7	8	Strongly Agree 9
★	★	★	★	★	★	★	★	★

* 156. Haemoptysis

Strongly Disagree 1	2	3	4	5	6	7	8	Strongly Agree 9
★	★	★	★	★	★	★	★	★

* 157. Increased or purulent sputum

Strongly Disagree 1	2	3	4	5	6	7	8	Strongly Agree 9
★	★	★	★	★	★	★	★	★

* 158. Persistent fatigue

Strongly Disagree 1	2	3	4	5	6	7	8	Strongly Agree 9

* 159. Increased frequency of exacerbations

Strongly Disagree 1	2	3	4	5	6	7	8	Strongly Agree 9

* 160. Fever

Strongly Disagree 1	2	3	4	5	6	7	8	Strongly Agree 9

* 161. Hoarseness

Strongly Disagree 1	2	3	4	5	6	7	8	Strongly Agree 9

* 162. GERD

Strongly Disagree 1	2	3	4	5	6	7	8	Strongly Agree 9

* 163. Decline in FEV1 of >20%

Strongly Disagree 1	2	3	4	5	6	7	8	Strongly Agree 9

* 164. Shortness of breath

Strongly Disagree 1	2	3	4	5	6	7	8	Strongly Agree 9

* 165. Combination of ≥2 or more symptoms outlined above

Strongly Disagree 1	2	3	4	5	6	7	8	Strongly Agree 9
★	★	★	★	★	★	★	★	★

* 166. Radiology suggestive of NTM

Strongly Disagree 1	2	3	4	5	6	7	8	Strongly Agree 9
★	★	★	★	★	★	★	★	★

IMMUNOSUPPRESSED PATIENTS AND MEDICATIONS

Which of the following medications patients who are immunosuppressed are taking would make you consider testing for NTM?

Patients in receipt of…

* 167. Macrolide therapy

Strongly Disagree 1	2	3	4	5	6	7	8	Strongly Agree 9
★	★	★	★	★	★	★	★	★

* 168. Any additional considerations? E.g. dose, type of macrolide, duration of use…

* 169. Inhaled corticosteroids

Strongly Disagree 1	2	3	4	5	6	7	8	Strongly Agree 9
★	★	★	★	★	★	★	★	★

* 170. Any additional considerations? E.g. dose, duration of use, specific steroids?

* 171. Oral corticosteroids

Strongly Disagree 1	2	3	4	5	6	7	8	Strongly Agree 9
★	★	★	★	★	★	★	★	★

* 172. Any additional considerations? E.g. dose, duration of use, specific steroids?

* 173. Monoclonal antibodies

Strongly Disagree 1	2	3	4	5	6	7	8	Strongly Agree 9
★	★	★	★	★	★	★	★	★

* 174. Any additional considerations? E.g. dose, duration of use, specific medications over others?

* 175. Biologic therapy e.g. immunomodulators

Strongly Disagree 1	2	3	4	5	6	7	8	Strongly Agree 9
★	★	★	★	★	★	★	★	★

* 176. Any additional considerations? E.g. dose, duration of use, specific medications over others?

* 177. IV corticosteroids

Strongly Disagree 1	2	3	4	5	6	7	8	Strongly Agree 9
★	★	★	★	★	★	★	★	★

* 178. Any additional considerations? E.g. dose, duration of use, specific steroids?

* 179. Immunosuppressant therapy

Strongly Disagree 1	2	3	4	5	6	7	8	Strongly Agree 9
★	★	★	★	★	★	★	★	★

* 180. Any additional considerations? E.g. dose, duration of use, specific medications over others?

* 181. Other medications not indicated above – please list

POST-TRANSPLANT PATIENTS

In patients who have undergone an organ transplant which symptoms would prompt you to test for NTM?

* 182. No symptoms

Strongly Disagree 1	2	3	4	5	6	7	8	Strongly Agree 9
☆	☆	☆	☆	☆	☆	☆	☆	☆

* 183. Persistent cough

Strongly Disagree 1	2	3	4	5	6	7	8	Strongly Agree 9
☆	☆	☆	☆	☆	☆	☆	☆	☆

* 184. Weight loss for no clear reason

Strongly Disagree 1	2	3	4	5	6	7	8	Strongly Agree 9
☆	☆	☆	☆	☆	☆	☆	☆	☆

* 185. Night sweats

Strongly Disagree 1	2	3	4	5	6	7	8	Strongly Agree 9
☆	☆	☆	☆	☆	☆	☆	☆	☆

* 186. Haemoptysis

Strongly Disagree 1	2	3	4	5	6	7	8	Strongly Agree 9
☆	☆	☆	☆	☆	☆	☆	☆	☆

* 187. Increased or purulent sputum

Strongly Disagree 1	2	3	4	5	6	7	8	Strongly Agree 9
☆	☆	☆	☆	☆	☆	☆	☆	☆

* 188. Persistent fatigue

Strongly Disagree 1	2	3	4	5	6	7	8	Strongly Agree 9

* 189. Increased frequency of exacerbations

Strongly Disagree 1	2	3	4	5	6	7	8	Strongly Agree 9

* 190. Fever

Strongly Disagree 1	2	3	4	5	6	7	8	Strongly Agree 9

* 191. Hoarseness

Strongly Disagree 1	2	3	4	5	6	7	8	Strongly Agree 9

* 192. GERD

Strongly Disagree 1	2	3	4	5	6	7	8	Strongly Agree 9

* 193. Decline in FEV1 of >20%

Strongly Disagree 1	2	3	4	5	6	7	8	Strongly Agree 9

* 194. Shortness of breath

Strongly Disagree 1	2	3	4	5	6	7	8	Strongly Agree 9

* 195. Combination of ≥2 or more symptoms outlined above

Strongly Disagree 1	2	3	4	5	6	7	8	Strongly Agree 9
☆	☆	☆	☆	☆	☆	☆	☆	☆

* 196. Radiology suggestive of NTM

Strongly Disagree 1	2	3	4	5	6	7	8	Strongly Agree 9
☆	☆	☆	☆	☆	☆	☆	☆	☆

POST-TRANSPLANT PATIENTS AND MEDICATIONS

Which of the following medications post-transplant patients are taking would make you consider testing for NTM?

Patients in receipt of…

* 197. Macrolide therapy

Strongly Disagree 1	2	3	4	5	6	7	8	Strongly Agree 9
★	★	★	★	★	★	★	★	★

* 198. Any additional considerations? E.g. dose, type of macrolide, duration of use…

* 199. Inhaled corticosteroids

Strongly Disagree 1	2	3	4	5	6	7	8	Strongly Agree 9
★	★	★	★	★	★	★	★	★

* 200. Any additional considerations? E.g. dose, duration of use, specific steroids?

* 201. Oral corticosteroids

Strongly Disagree 1	2	3	4	5	6	7	8	Strongly Agree 9
★	★	★	★	★	★	★	★	★

* 202. Any additional considerations? E.g. dose, duration of use, specific steroids?

* 203. Monoclonal antibodies

Strongly Disagree 1	2	3	4	5	6	7	8	Strongly Agree 9
★	★	★	★	★	★	★	★	★

* 204. Any additional considerations? E.g. dose, duration of use, specific medications over others?

* 205. Biologic therapy e.g. immunomodulators

Strongly Disagree 1	2	3	4	5	6	7	8	Strongly Agree 9
★	★	★	★	★	★	★	★	★

* 206. Any additional considerations? E.g. dose, duration of use, specific medications over others?

* 207. IV corticosteroids

Strongly Disagree 1	2	3	4	5	6	7	8	Strongly Agree 9
★	★	★	★	★	★	★	★	★

* 208. Any additional considerations? E.g. dose, duration of use, specific steroids?

* 209. Immunosuppressant therapy

Strongly Disagree 1	2	3	4	5	6	7	8	Strongly Agree 9
★	★	★	★	★	★	★	★	★

* 210. Any additional considerations? E.g. dose, duration of use, specific medications over others?

* 211. Other medications not indicated above – please list

PATIENTS WITH RENAL DISEASE

In patients who have renal impairment/renal disease which symptoms would prompt you to test for NTM?

* 212. No symptoms

Strongly Disagree 1	2	3	4	5	6	7	8	Strongly Agree 9
☆	☆	☆	☆	☆	☆	☆	☆	☆

* 213. Persistent cough

Strongly Disagree 1	2	3	4	5	6	7	8	Strongly Agree 9
☆	☆	☆	☆	☆	☆	☆	☆	☆

* 214. Weight loss for no clear reason

Strongly Disagree 1	2	3	4	5	6	7	8	Strongly Agree 9
☆	☆	☆	☆	☆	☆	☆	☆	☆

* 215. Night sweats

Strongly Disagree 1	2	3	4	5	6	7	8	Strongly Agree 9
☆	☆	☆	☆	☆	☆	☆	☆	☆

* 216. Haemoptysis

Strongly Disagree 1	2	3	4	5	6	7	8	Strongly Agree 9
☆	☆	☆	☆	☆	☆	☆	☆	☆

* 217. Increased or purulent sputum

Strongly Disagree 1	2	3	4	5	6	7	8	Strongly Agree 9
☆	☆	☆	☆	☆	☆	☆	☆	☆

* 218. Persistent fatigue

Strongly Disagree 1	2	3	4	5	6	7	8	Strongly Agree 9
★	★	★	★	★	★	★	★	★

* 219. Increased frequency of exacerbations

Strongly Disagree 1	2	3	4	5	6	7	8	Strongly Agree 9
★	★	★	★	★	★	★	★	★

* 220. Fever

Strongly Disagree 1	2	3	4	5	6	7	8	Strongly Agree 9
★	★	★	★	★	★	★	★	★

* 221. Hoarseness

Strongly Disagree 1	2	3	4	5	6	7	8	Strongly Agree 9
★	★	★	★	★	★	★	★	★

* 222. GERD

Strongly Disagree 1	2	3	4	5	6	7	8	Strongly Agree 9
★	★	★	★	★	★	★	★	★

* 223. Decline in FEV1 of >20%

Strongly Disagree 1	2	3	4	5	6	7	8	Strongly Agree 9
★	★	★	★	★	★	★	★	★

* 224. Shortness of breath

Strongly Disagree 1	2	3	4	5	6	7	8	Strongly Agree 9
★	★	★	★	★	★	★	★	★

* 225. Combination of ≥2 or more symptoms outlined above

Strongly Disagree 1	2	3	4	5	6	7	8	Strongly Agree 9
☆	☆	☆	☆	☆	☆	☆	☆	☆

* 226. Radiology suggestive of NTM

Strongly Disagree 1	2	3	4	5	6	7	8	Strongly Agree 9
☆	☆	☆	☆	☆	☆	☆	☆	☆

PATIENTS WITH RENAL DISEASE AND MEDICATIONS

Which of the following medications in patients with renal disease are taking would make you consider testing for NTM?

Patients in receipt of…

* 227. Macrolide therapy

Strongly Disagree 1	2	3	4	5	6	7	8	Strongly Agree 9
★	★	★	★	★	★	★	★	★

* 228. Any additional considerations? E.g. dose, type of macrolide, duration of use…

* 229. Inhaled corticosteroids

Strongly Disagree 1	2	3	4	5	6	7	8	Strongly Agree 9
★	★	★	★	★	★	★	★	★

* 230. Any additional considerations? E.g. dose, duration of use, specific steroids?

* 231. Oral corticosteroids

Strongly Disagree 1	2	3	4	5	6	7	8	Strongly Agree 9
★	★	★	★	★	★	★	★	★

* 232. Any additional considerations? E.g. dose, duration of use, specific steroids?

* 233. Monoclonal antibodies

Strongly Disagree 1	2	3	4	5	6	7	8	Strongly Agree 9
★	★	★	★	★	★	★	★	★

* 234. Any additional considerations? E.g. dose, duration of use, specific medications over others?

* 235. Biologic therapy e.g. immunomodulators

Strongly Disagree 1	2	3	4	5	6	7	8	Strongly Agree 9
★	★	★	★	★	★	★	★	★

* 236. Any additional considerations? E.g. dose, duration of use, specific medications over others?

* 237. IV corticosteroids

Strongly Disagree 1	2	3	4	5	6	7	8	Strongly Agree 9
★	★	★	★	★	★	★	★	★

* 238. Any additional considerations? E.g. dose, duration of use, specific steroids?

* 239. Immunosuppressant therapy

Strongly Disagree 1	2	3	4	5	6	7	8	Strongly Agree 9
★	★	★	★	★	★	★	★	★

* 240. Any additional considerations? E.g. dose, duration of use, specific medications over others?

* 241. Other medications not indicated above – please list

PATIENTS WITH DIABETES

In patients with diabetes which symptoms would prompt you to test for NTM?

* 242. No Symptoms

Strongly Disagree 1	2	3	4	5	6	7	8	Strongly Agree 9
☆	☆	☆	☆	☆	☆	☆	☆	☆

* 243. Persistent cough

Strongly Disagree 1	2	3	4	5	6	7	8	Strongly Agree 9
☆	☆	☆	☆	☆	☆	☆	☆	☆

* 244. Weight loss for no clear reason

Strongly Disagree 1	2	3	4	5	6	7	8	Strongly Agree 9
☆	☆	☆	☆	☆	☆	☆	☆	☆

* 245. Night sweats

Strongly Disagree 1	2	3	4	5	6	7	8	Strongly Agree 9
☆	☆	☆	☆	☆	☆	☆	☆	☆

* 246. Haemoptysis

Strongly Disagree 1	2	3	4	5	6	7	8	Strongly Agree 9
☆	☆	☆	☆	☆	☆	☆	☆	☆

* 247. Increased or purulent sputum

Strongly Disagree 1	2	3	4	5	6	7	8	Strongly Agree 9
☆	☆	☆	☆	☆	☆	☆	☆	☆

* 248. Persistent fatigue

Strongly Disagree 1	2	3	4	5	6	7	8	Strongly Agree 9
☆	☆	☆	☆	☆	☆	☆	☆	☆

* 249. Increased frequency of exacerbations

Strongly Disagree 1	2	3	4	5	6	7	8	Strongly Agree 9
☆	☆	☆	☆	☆	☆	☆	☆	☆

* 250. Fever

Strongly Disagree 1	2	3	4	5	6	7	8	Strongly Agree 9
☆	☆	☆	☆	☆	☆	☆	☆	☆

* 251. Hoarseness

Strongly Disagree 1	2	3	4	5	6	7	8	Strongly Agree 9
☆	☆	☆	☆	☆	☆	☆	☆	☆

* 252. GERD

Strongly Disagree 1	2	3	4	5	6	7	8	Strongly Agree 9
☆	☆	☆	☆	☆	☆	☆	☆	☆

* 253. Decline in FEV1 of >20%

Strongly Disagree 1	2	3	4	5	6	7	8	Strongly Agree 9
☆	☆	☆	☆	☆	☆	☆	☆	☆

* 254. Shortness of breath

Strongly Disagree 1	2	3	4	5	6	7	8	Strongly Agree 9
☆	☆	☆	☆	☆	☆	☆	☆	☆

* 255. Combination of ≥2 or more symptoms outlined above

Strongly Disagree 1	2	3	4	5	6	7	8	Strongly Agree 9
★	★	★	★	★	★	★	★	★

* 256. Radiology suggestive of NTM

Strongly Disagree 1	2	3	4	5	6	7	8	Strongly Agree 9
★	★	★	★	★	★	★	★	★

PATIENTS WITH DIABETES AND MEDICATIONS

Which of the following medications patients with diabetes are taking would make you consider testing for NTM?

Patients in receipt of…

* 257. Macrolide therapy

Strongly Disagree 1	2	3	4	5	6	7	8	Strongly Agree 9
★	★	★	★	★	★	★	★	★

* 258. Any additional considerations? E.g. dose, type of macrolide, duration of use…

* 259. Inhaled corticosteroids

Strongly Disagree 1	2	3	4	5	6	7	8	Strongly Agree 9
★	★	★	★	★	★	★	★	★

* 260. Any additional considerations? E.g. dose, duration of use, specific steroids?

* 261. Oral corticosteroids

Strongly Disagree 1	2	3	4	5	6	7	8	Strongly Agree 9
★	★	★	★	★	★	★	★	★

* 262. Any additional considerations? E.g. dose, duration of use, specific steroids?

* 263. Monoclonal antibodies

Strongly Disagree 1	2	3	4	5	6	7	8	Strongly Agree 9
★	★	★	★	★	★	★	★	★

* 264. Any additional considerations? E.g. dose, duration of use, specific medications over others?

* 265. Biologic therapy e.g. immunomodulators

Strongly Disagree 1	2	3	4	5	6	7	8	Strongly Agree 9
★	★	★	★	★	★	★	★	★

* 266. Any additional considerations? E.g. dose, duration of use, specific medications over others?

* 267. IV corticosteroids

Strongly Disagree 1	2	3	4	5	6	7	8	Strongly Agree 9
★	★	★	★	★	★	★	★	★

* 268. Any additional considerations? E.g. dose, duration of use, specific steroids?

* 269. Immunosuppressant therapy

Strongly Disagree 1	2	3	4	5	6	7	8	Strongly Agree 9
★	★	★	★	★	★	★	★	★

* 270. Any additional considerations? E.g. dose, duration of use, specific medications over others?

* 271. Other medications not indicated above – please list

CARDIOVASCULAR DISEASE

In patients with cardiovascular disease which symptoms would prompt you to test for NTM?

* 272. No symptoms

Strongly Disagree 1	2	3	4	5	6	7	8	Strongly Agree 9
★	★	★	★	★	★	★	★	★

* 273. Persistent cough

Strongly Disagree 1	2	3	4	5	6	7	8	Strongly Agree 9
★	★	★	★	★	★	★	★	★

* 274. Weight loss for no clear reason

Strongly Disagree 1	2	3	4	5	6	7	8	Strongly Agree 9
★	★	★	★	★	★	★	★	★

* 275. Night sweats

Strongly Disagree 1	2	3	4	5	6	7	8	Strongly Agree 9
★	★	★	★	★	★	★	★	★

* 276. Haemoptysis

Strongly Disagree 1	2	3	4	5	6	7	8	Strongly Agree 9
★	★	★	★	★	★	★	★	★

* 277. Increased or purulent sputum

Strongly Disagree 1	2	3	4	5	6	7	8	Strongly Agree 9
★	★	★	★	★	★	★	★	★

* 278. Persistant fatigue

Strongly Disagree 1	2	3	4	5	6	7	8	Strongly Agree 9

* 279. Increased frequency of exacerbations

Strongly Disagree 1	2	3	4	5	6	7	8	Strongly Agree 9

* 280. Fever

Strongly Disagree 1	2	3	4	5	6	7	8	Strongly Agree 9

* 281. Hoarseness

Strongly Disagree 1	2	3	4	5	6	7	8	Strongly Agree 9

* 282. GERD

Strongly Disagree 1	2	3	4	5	6	7	8	Strongly Agree 9

* 283. Decline in FEV1 of >20%

Strongly Disagree 1	2	3	4	5	6	7	8	Strongly Agree 9

* 284. Shortness of breath

Strongly Disagree 1	2	3	4	5	6	7	8	Strongly Agree 9

* 285. Combination of ≥2 or more symptoms outlined above

Strongly Disagree 1	2	3	4	5	6	7	8	Strongly Agree 9
☆	☆	☆	☆	☆	☆	☆	☆	☆

* 286. Radiology suggestive of NTM

Strongly Disagree 1	2	3	4	5	6	7	8	Strongly Agree 9
☆	☆	☆	☆	☆	☆	☆	☆	☆

CARDIOVASCULAR DISEASE AND MEDICATION

Which of the following medications patients with cardiovascular disease are taking would make you consider testing for NTM?

Patients in receipt of...

* 287. Macrolide therapy

Strongly Disagree 1	2	3	4	5	6	7	8	Strongly Agree 9
☆	☆	☆	☆	☆	☆	☆	☆	☆

* 288. Any additional considerations? E.g. dose, type of macrolide, duration of use...

* 289. Inhaled corticosteroids

Strongly Disagree 1	2	3	4	5	6	7	8	Strongly Agree 9
☆	☆	☆	☆	☆	☆	☆	☆	☆

* 290. Any additional considerations? E.g. dose, duration of use, specific steroids?

* 291. Oral corticosteroids

Strongly Disagree 1	2	3	4	5	6	7	8	Strongly Agree 9
☆	☆	☆	☆	☆	☆	☆	☆	☆

* 292. Any additional considerations? E.g. dose, duration of use, specific steroids?

* 293. Monoclonal antibodies

Strongly Disagree 1	2	3	4	5	6	7	8	Strongly Agree 9
★	★	★	★	★	★	★	★	★

* 294. Any additional considerations? E.g. dose, duration of use, specific medications over others?

* 295. Biologic therapy e.g. immunomodulators

Strongly Disagree 1	2	3	4	5	6	7	8	Strongly Agree 9
★	★	★	★	★	★	★	★	★

* 296. Any additional considerations? E.g. dose, duration of use, specific medications over others?

* 297. IV corticosteroids

Strongly Disagree 1	2	3	4	5	6	7	8	Strongly Agree 9
★	★	★	★	★	★	★	★	★

* 298. Any additional considerations? E.g. dose, duration of use, specific steroids?

* 299. Immunosuppressant therapy

Strongly Disagree 1	2	3	4	5	6	7	8	Strongly Agree 9
★	★	★	★	★	★	★	★	★

* 300. Any additional considerations? E.g. dose, duration of use, specific steroids?

* 301. Other medications not indicated above – please list

RHEUMATOID ARTHRITIS

In patients with rheumatoid arthritis which symptoms would prompt you to test for NTM?

* 302. No Symptoms

Strongly Disagree 1	2	3	4	5	6	7	8	Strongly Agree 9
★	★	★	★	★	★	★	★	★

* 303. Persistent cough

Strongly Disagree 1	2	3	4	5	6	7	8	Strongly Agree 9
★	★	★	★	★	★	★	★	★

* 304. Weight loss for no clear reason

Strongly Disagree 1	2	3	4	5	6	7	8	Strongly Agree 9
★	★	★	★	★	★	★	★	★

* 305. Night sweats

Strongly Disagree 1	2	3	4	5	6	7	8	Strongly Agree 9
★	★	★	★	★	★	★	★	★

* 306. Haemoptysis

Strongly Disagree 1	2	3	4	5	6	7	8	Strongly Agree 9
★	★	★	★	★	★	★	★	★

* 307. Increased or purulent sputum

Strongly Disagree 1	2	3	4	5	6	7	8	Strongly Agree 9
★	★	★	★	★	★	★	★	★

* 308. Persistent fatigue

Strongly Disagree 1	2	3	4	5	6	7	8	Strongly Agree 9
☆	☆	☆	☆	☆	☆	☆	☆	☆

* 309. Increased frequency of exacerbations

Strongly Disagree 1	2	3	4	5	6	7	8	Strongly Agree 9
☆	☆	☆	☆	☆	☆	☆	☆	☆

* 310. Fever

Strongly Disagree 1	2	3	4	5	6	7	8	Strongly Agree 9
☆	☆	☆	☆	☆	☆	☆	☆	☆

* 311. Hoarseness

Strongly Disagree 1	2	3	4	5	6	7	8	Strongly Agree 9
☆	☆	☆	☆	☆	☆	☆	☆	☆

* 312. GERD

Strongly Disagree 1	2	3	4	5	6	7	8	Strongly Agree 9
☆	☆	☆	☆	☆	☆	☆	☆	☆

* 313. Decline in FEV1 of >20%

Strongly Disagree 1	2	3	4	5	6	7	8	Strongly Agree 9
☆	☆	☆	☆	☆	☆	☆	☆	☆

* 314. Shortness of breath

Strongly Disagree 1	2	3	4	5	6	7	8	Strongly Agree 9
☆	☆	☆	☆	☆	☆	☆	☆	☆

* 315. Combination of ≥2 or more symptoms outlined above

Strongly Disagree 1	2	3	4	5	6	7	8	Strongly Agree 9
☆	☆	☆	☆	☆	☆	☆	☆	☆

* 316. Radiology suggestive of NTM

Strongly Disagree 1	2	3	4	5	6	7	8	Strongly Agree 9
☆	☆	☆	☆	☆	☆	☆	☆	☆

RHEUMATOID ARTHRITIS AND MEDICATIONS

In patients with rheumatoid arthritis which symptoms would prompt you to test for NTM?

* 317. Macrolide therapy

Strongly Disagree 1	2	3	4	5	6	7	8	Strongly Agree 9
★	★	★	★	★	★	★	★	★

* 318. Any additional considerations? E.g. dose, type of macrolide, duration of use...

* 319. Inhaled corticosteroids

Strongly Disagree 1	2	3	4	5	6	7	8	Strongly Agree 9
★	★	★	★	★	★	★	★	★

* 320. Any additional considerations? E.g. dose, duration of use, specific steroids?

* 321. Oral corticosteroids

Strongly Disagree 1	2	3	4	5	6	7	8	Strongly Agree 9
★	★	★	★	★	★	★	★	★

* 322. Any additional considerations? E.g. dose, duration of use, specific steroids?

* 323. Monoclonal antibodies

Strongly Disagree 1	2	3	4	5	6	7	8	Strongly Agree 9
★	★	★	★	★	★	★	★	★

* 324. Any additional considerations? E.g. dose, duration of use, specific medications over others?

* 325. Biologic therapy e.g. immunomodulators

Strongly Disagree 1	2	3	4	5	6	7	8	Strongly Agree 9
☆	☆	☆	☆	☆	☆	☆	☆	☆

* 326. Any additional considerations? E.g. dose, duration of use, specific medications over others?

* 327. IV corticosteroids

Strongly Disagree 1	2	3	4	5	6	7	8	Strongly Agree 9
☆	☆	☆	☆	☆	☆	☆	☆	☆

* 328. Any additional considerations? E.g. dose, duration of use, specific steroids?

* 329. Immunosuppressant therapy

Strongly Disagree 1	2	3	4	5	6	7	8	Strongly Agree 9
☆	☆	☆	☆	☆	☆	☆	☆	☆

* 330. Any additional considerations? E.g. dose, duration of use, specific medications over others?

* 331. Other medications not indicated above – please list

CANCER

In patients with active cancer which symptoms would prompt you to test for NTM?

* 332. No symptoms

Strongly Disagree 1	2	3	4	5	6	7	8	Strongly Agree 9
★	★	★	★	★	★	★	★	★

* 333. Persistent cough

Strongly Disagree 1	2	3	4	5	6	7	8	Strongly Agree 9
★	★	★	★	★	★	★	★	★

* 334. Weight loss for no clear reason

Strongly Disagree 1	2	3	4	5	6	7	8	Strongly Agree 9
★	★	★	★	★	★	★	★	★

* 335. Night sweats

Strongly Disagree 1	2	3	4	5	6	7	8	Strongly Agree 9
★	★	★	★	★	★	★	★	★

* 336. Haemoptysis

Strongly Disagree 1	2	3	4	5	6	7	8	Strongly Agree 9
★	★	★	★	★	★	★	★	★

* 337. Increased or purulent sputum

Strongly Disagree 1	2	3	4	5	6	7	8	Strongly Agree 9
★	★	★	★	★	★	★	★	★

* 338. Persistent fatigue

Strongly Disagree 1	2	3	4	5	6	7	8	Strongly Agree 9
★	★	★	★	★	★	★	★	★

* 339. Increased frequency of exacerbations

Strongly Disagree 1	2	3	4	5	6	7	8	Strongly Agree 9
★	★	★	★	★	★	★	★	★

* 340. Fever

Strongly Disagree 1	2	3	4	5	6	7	8	Strongly Agree 9
★	★	★	★	★	★	★	★	★

* 341. Hoarseness

Strongly Disagree 1	2	3	4	5	6	7	8	Strongly Agree 9
★	★	★	★	★	★	★	★	★

* 342. GERD

Strongly Disagree 1	2	3	4	5	6	7	8	Strongly Agree 9
★	★	★	★	★	★	★	★	★

* 343. Decline in FEV1 of >20%

Strongly Disagree 1	2	3	4	5	6	7	8	Strongly Agree 9
★	★	★	★	★	★	★	★	★

* 344. Shortness of breath

Strongly Disagree 1	2	3	4	5	6	7	8	Strongly Agree 9
★	★	★	★	★	★	★	★	★

* 345. Combination of ≥2 or more symptoms outlined above

Strongly Disagree 1	2	3	4	5	6	7	8	Strongly Agree 9
★	★	★	★	★	★	★	★	★

* 346. Radiology suggestive of NTM

Strongly Disagree 1	2	3	4	5	6	7	8	Strongly Agree 9
★	★	★	★	★	★	★	★	★

CANCER AND MEDICATIONS

Which of the following medications patients with cancer are taking would make you consider testing for NTM?

Patients in receipt of…

* 347. Macrolide therapy

Strongly Disagree 1	2	3	4	5	6	7	8	Strongly Agree 9
☆	☆	☆	☆	☆	☆	☆	☆	☆

* 348. Any additional considerations? E.g. dose, type of macrolide, duration of use…

* 349. Inhaled corticosteroids

Strongly Disagree 1	2	3	4	5	6	7	8	Strongly Agree 9
☆	☆	☆	☆	☆	☆	☆	☆	☆

* 350. Any additional considerations? E.g. dose, duration of use, specific steroids?Any additional considerations? E.g. dose, duration of use, specific steroids?

* 351. Oral corticosteroids

Strongly Disagree 1	2	3	4	5	6	7	8	Strongly Agree 9
☆	☆	☆	☆	☆	☆	☆	☆	☆

* 352. Any additional considerations? E.g. dose, duration of use, specific steroids?

* 353. Monoclonal antibodies

Strongly Disagree 1	2	3	4	5	6	7	8	Strongly Agree 9
★	★	★	★	★	★	★	★	★

* 354. Any additional considerations? E.g. dose, duration of use, specific medications over others?

* 355. Biologic therapy e.g. immunomodulators

Strongly Disagree 1	2	3	4	5	6	7	8	Strongly Agree 9
★	★	★	★	★	★	★	★	★

* 356. Any additional considerations? E.g. dose, duration of use, specific medications over others?

* 357. IV corticosteroids

Strongly Disagree 1	2	3	4	5	6	7	8	Strongly Agree 9
★	★	★	★	★	★	★	★	★

* 358. Any additional considerations? E.g. dose, duration of use, specific steroids?

* 359. Immunosuppressant therapy

Strongly Disagree 1	2	3	4	5	6	7	8	Strongly Agree 9
★	★	★	★	★	★	★	★	★

* 360. Any additional considerations? E.g. dose, duration of use, specific medications over others?

* 361. Other medications not indicated above – please list

NO BACKGROUND RESPIRATORY COMORBIDITY

In patients with no background respiratory comorbidities which symptoms would prompt you to test for NTM?

* 362. No symptoms

Strongly Disagree 1	2	3	4	5	6	7	8	Strongly Agree 9
☆	☆	☆	☆	☆	☆	☆	☆	☆

* 363. Persistent cough

Strongly Disagree 1	2	3	4	5	6	7	8	Strongly Agree 9
☆	☆	☆	☆	☆	☆	☆	☆	☆

* 364. Weight loss for no clear reason

Strongly Disagree 1	2	3	4	5	6	7	8	Strongly Agree 9
☆	☆	☆	☆	☆	☆	☆	☆	☆

* 365. Night sweats

Strongly Disagree 1	2	3	4	5	6	7	8	Strongly Agree 9
☆	☆	☆	☆	☆	☆	☆	☆	☆

* 366. Haemoptysis

Strongly Disagree 1	2	3	4	5	6	7	8	Strongly Agree 9
☆	☆	☆	☆	☆	☆	☆	☆	☆

* 367. Increased or purulent sputum

Strongly Disagree 1	2	3	4	5	6	7	8	Strongly Agree 9
☆	☆	☆	☆	☆	☆	☆	☆	☆

* 368. Persistent fatigue

Strongly Disagree 1	2	3	4	5	6	7	8	Strongly Agree 9
☆	☆	☆	☆	☆	☆	☆	☆	☆

* 369. Increased frequency of exacerbations

Strongly Disagree 1	2	3	4	5	6	7	8	Strongly Agree 9
☆	☆	☆	☆	☆	☆	☆	☆	☆

* 370. Fever

Strongly Disagree 1	2	3	4	5	6	7	8	Strongly Agree 9
☆	☆	☆	☆	☆	☆	☆	☆	☆

* 371. Hoarseness

Strongly Disagree 1	2	3	4	5	6	7	8	Strongly Agree 9
☆	☆	☆	☆	☆	☆	☆	☆	☆

* 372. GERD

Strongly Disagree 1	2	3	4	5	6	7	8	Strongly Agree 9
☆	☆	☆	☆	☆	☆	☆	☆	☆

* 373. Decline in FEV1 of >20%

Strongly Disagree 1	2	3	4	5	6	7	8	Strongly Agree 9
☆	☆	☆	☆	☆	☆	☆	☆	☆

* 374. Shortness of breath

Strongly Disagree 1	2	3	4	5	6	7	8	Strongly Agree 9
☆	☆	☆	☆	☆	☆	☆	☆	☆

* 375. Combination of ≥2 or more symptoms outlined above

Strongly Disagree 1	2	3	4	5	6	7	8	Strongly Agree 9
☆	☆	☆	☆	☆	☆	☆	☆	☆

* 376. Radiology suggestive of NTM

Strongly Disagree 1	2	3	4	5	6	7	8	Strongly Agree 9
☆	☆	☆	☆	☆	☆	☆	☆	☆

ALLERGIC RHINITIS AND MEDICATIONS

Which of the following medications patients with allergic rhinitis are taking would make you consider testing for NTM?

Patients in receipt of…

* 377. Macrolide therapy

Strongly Disagree 1	2	3	4	5	6	7	8	Strongly Agree 9
☆	☆	☆	☆	☆	☆	☆	☆	☆

* 378. Any additional considerations? E.g. dose, type of macrolide, duration of use…

* 379. Inhaled corticosteroids

Strongly Disagree 1	2	3	4	5	6	7	8	Strongly Agree 9
☆	☆	☆	☆	☆	☆	☆	☆	☆

* 380. Any additional considerations? E.g. dose, duration of use, specific steroids?

* 381. Oral corticosteroids

Strongly Disagree 1	2	3	4	5	6	7	8	Strongly Agree 9
☆	☆	☆	☆	☆	☆	☆	☆	☆

* 382. Any additional considerations? E.g. dose, duration of use, specific steroids?

* 383. Monoclonal antibodies

Strongly Disagree 1	2	3	4	5	6	7	8	Strongly Agree 9
☆	☆	☆	☆	☆	☆	☆	☆	☆

* 384. Any additional considerations? E.g. dose, duration of use, specific medications over others?

* 385. Biologic therapy e.g. immunomodulators

Strongly Disagree 1	2	3	4	5	6	7	8	Strongly Agree 9
★	★	★	★	★	★	★	★	★

* 386. Any additional considerations? E.g. dose, duration of use, specific medications over others?

* 387. IV corticosteroids

Strongly Disagree 1	2	3	4	5	6	7	8	Strongly Agree 9
★	★	★	★	★	★	★	★	★

* 388. Any additional considerations? E.g. dose, duration of use, specific steroids?

* 389. Immunosuppressant therapy

Strongly Disagree 1	2	3	4	5	6	7	8	Strongly Agree 9
★	★	★	★	★	★	★	★	★

* 390. Any additional considerations? E.g. dose, duration of use, specific medications over others?

* 391. Other medications not indicated above – please list

INTERSTITIAL LUNG DISEASE

In patients with ILD which symptoms would prompt you to test for NTM?

* 392. No Symptoms

Strongly Disagree 1	2	3	4	5	6	7	8	Strongly Agree 9
☆	☆	☆	☆	☆	☆	☆	☆	☆

* 393. Persistent cough

Strongly Disagree 1	2	3	4	5	6	7	8	Strongly Agree 9
☆	☆	☆	☆	☆	☆	☆	☆	☆

* 394. Weight loss for no clear reason

Strongly Disagree 1	2	3	4	5	6	7	8	Strongly Agree 9
☆	☆	☆	☆	☆	☆	☆	☆	☆

* 395. Night sweats

Strongly Disagree 1	2	3	4	5	6	7	8	Strongly Agree 9
☆	☆	☆	☆	☆	☆	☆	☆	☆

* 396. Haemoptysis

Strongly Disagree 1	2	3	4	5	6	7	8	Strongly Agree 9
☆	☆	☆	☆	☆	☆	☆	☆	☆

* 397. Increased or purulent sputum

Strongly Disagree 1	2	3	4	5	6	7	8	Strongly Agree 9
☆	☆	☆	☆	☆	☆	☆	☆	☆

* 398. Persistent fatigue

Strongly Disagree 1	2	3	4	5	6	7	8	Strongly Agree 9
☆	☆	☆	☆	☆	☆	☆	☆	☆

* 399. Increased frequency of exacerbations

Strongly Disagree 1	2	3	4	5	6	7	8	Strongly Agree 9
☆	☆	☆	☆	☆	☆	☆	☆	☆

* 400. Fever

Strongly Disagree 1	2	3	4	5	6	7	8	Strongly Agree 9
☆	☆	☆	☆	☆	☆	☆	☆	☆

* 401. Hoarseness

Strongly Disagree 1	2	3	4	5	6	7	8	Strongly Agree 9
☆	☆	☆	☆	☆	☆	☆	☆	☆

* 402. GERD

Strongly Disagree 1	2	3	4	5	6	7	8	Strongly Agree 9
☆	☆	☆	☆	☆	☆	☆	☆	☆

* 403. Decline in FEV1 of >20%

Strongly Disagree 1	2	3	4	5	6	7	8	Strongly Agree 9
☆	☆	☆	☆	☆	☆	☆	☆	☆

* 404. Shortness of breath

Strongly Disagree 1	2	3	4	5	6	7	8	Strongly Agree 9
☆	☆	☆	☆	☆	☆	☆	☆	☆

* 405. Combination of ≥2 or more symptoms outlined above

Strongly Disagree 1	2	3	4	5	6	7	8	Strongly Agree 9
★	★	★	★	★	★	★	★	★

* 406. Radiology suggestive of NTM

Strongly Disagree 1	2	3	4	5	6	7	8	Strongly Agree 9
★	★	★	★	★	★	★	★	★

INTERSTITIAL LUNG DISEASE AND MEDICATION

Which of the following medications patients with ILD are taking would make you consider testing for NTM?

Patients in receipt of…

* 407. Macrolide therapy

Strongly Disagree 1	2	3	4	5	6	7	8	Strongly Agree 9
☆	☆	☆	☆	☆	☆	☆	☆	☆

* 408. Any additional considerations? E.g. dose, type of macrolide, duration of use…

* 409. Inhaled corticosteroids

Strongly Disagree 1	2	3	4	5	6	7	8	Strongly Agree 9
☆	☆	☆	☆	☆	☆	☆	☆	☆

* 410. Any additional considerations? E.g. dose, duration of use, specific steroids?

* 411. Oral corticosteroids

Strongly Disagree 1	2	3	4	5	6	7	8	Strongly Agree 9
☆	☆	☆	☆	☆	☆	☆	☆	☆

* 412. Any additional considerations? E.g. dose, duration of use, specific steroids?

* 413. Monoclonal antibodies

Strongly Disagree 1	2	3	4	5	6	7	8	Strongly Agree 9
☆	☆	☆	☆	☆	☆	☆	☆	☆

* 414. Any additional considerations? E.g. dose, duration of use, specific medications over others?

* 415. Biologic therapy e.g. immunomodulators

Strongly Disagree 1	2	3	4	5	6	7	8	Strongly Agree 9
☆	☆	☆	☆	☆	☆	☆	☆	☆

* 416. Any additional considerations? E.g. dose, duration of use, specific medications over others?

* 417. IV corticosteroids

Strongly Disagree 1	2	3	4	5	6	7	8	Strongly Agree 9
☆	☆	☆	☆	☆	☆	☆	☆	☆

* 418. Any additional considerations? E.g. dose, duration of use, specific steroids?

* 419. Immunosuppressant therapy

Strongly Disagree 1	2	3	4	5	6	7	8	Strongly Agree 9
☆	☆	☆	☆	☆	☆	☆	☆	☆

* 420. Any additional considerations? E.g. dose, duration of use, specific medications over others?

* 421. Other medications not indicated above – please list

DECLINING LUNG FUNCTION (FEV1% PREDICTED)

In patients with worsening lung function which symptoms would prompt you to test for NTM?

* 422. No symptoms

Strongly Disagree 1	2	3	4	5	6	7	8	Strongly Agree 9
★	★	★	★	★	★	★	★	★

* 423. Persistent cough

Strongly Disagree 1	2	3	4	5	6	7	8	Strongly Agree 9
★	★	★	★	★	★	★	★	★

* 424. Weight loss for no clear reason

Strongly Disagree 1	2	3	4	5	6	7	8	Strongly Agree 9
★	★	★	★	★	★	★	★	★

* 425. Night sweats

Strongly Disagree 1	2	3	4	5	6	7	8	Strongly Agree 9
★	★	★	★	★	★	★	★	★

* 426. Haemoptysis

Strongly Disagree 1	2	3	4	5	6	7	8	Strongly Agree 9
★	★	★	★	★	★	★	★	★

* 427. Increased or purulent sputum

Strongly Disagree 1	2	3	4	5	6	7	8	Strongly Agree 9
★	★	★	★	★	★	★	★	★

* 428. Persistent fatigue

Strongly Disagree 1	2	3	4	5	6	7	8	Strongly Agree 9
★	★	★	★	★	★	★	★	★

* 429. Increased frequency of exacerbations

Strongly Disagree 1	2	3	4	5	6	7	8	Strongly Agree 9
★	★	★	★	★	★	★	★	★

* 430. Fever

Strongly Disagree 1	2	3	4	5	6	7	8	Strongly Agree 9
★	★	★	★	★	★	★	★	★

* 431. Hoarseness

Strongly Disagree 1	2	3	4	5	6	7	8	Strongly Agree 9
★	★	★	★	★	★	★	★	★

* 432. GERD

Strongly Disagree 1	2	3	4	5	6	7	8	Strongly Agree 9
★	★	★	★	★	★	★	★	★

* 433. Decline in FEV1 of >20%

Strongly Disagree 1	2	3	4	5	6	7	8	Strongly Agree 9
★	★	★	★	★	★	★	★	★

* 434. Shortness of breath

Strongly Disagree 1	2	3	4	5	6	7	8	Strongly Agree 9
★	★	★	★	★	★	★	★	★

* 435. Combination of ≥2 or more symptoms outlined above

Strongly Disagree 1	2	3	4	5	6	7	8	Strongly Agree 9
☆	☆	☆	☆	☆	☆	☆	☆	☆

* 436. Radiology suggestive of NTM

Strongly Disagree 1	2	3	4	5	6	7	8	Strongly Agree 9
☆	☆	☆	☆	☆	☆	☆	☆	☆

DECLINING LUNG FUNCTION (FEV1%) AND MEDICATIONS

Which of the following medications patients with declining lung function (FEV1%) are taking would make you consider testing for NTM?

Patients in receipt of…

* 437. Macrolide therapy

Strongly Disagree 1	2	3	4	5	6	7	8	Strongly Agree 9
☆	☆	☆	☆	☆	☆	☆	☆	☆

* 438. Any additional considerations? E.g. dose, type of macrolide, duration of use…

* 439. Inhaled corticosteroids

Strongly Disagree 1	2	3	4	5	6	7	8	Strongly Agree 9
☆	☆	☆	☆	☆	☆	☆	☆	☆

* 440. Any additional considerations? E.g. dose, duration of use, specific steroids?

* 441. Oral corticosteroids

Strongly Disagree 1	2	3	4	5	6	7	8	Strongly Agree 9
☆	☆	☆	☆	☆	☆	☆	☆	☆

* 442. Any additional considerations? E.g. dose, duration of use, specific steroids?

* 443. Monoclonal antibodies

Strongly Disagree 1	2	3	4	5	6	7	8	Strongly Agree 9
☆	☆	☆	☆	☆	☆	☆	☆	☆

* 444. Any additional considerations? E.g. dose, duration of use, specific medications over others?

* 445. Biologic therapy e.g. immunomodulators

Strongly Disagree 1	2	3	4	5	6	7	8	Strongly Agree 9
☆	☆	☆	☆	☆	☆	☆	☆	☆

* 446. Any additional considerations? E.g. dose, duration of use, specific medications over others?

* 447. IV corticosteroids

Strongly Disagree 1	2	3	4	5	6	7	8	Strongly Agree 9
☆	☆	☆	☆	☆	☆	☆	☆	☆

* 448. Any additional considerations? E.g. dose, duration of use, specific steroids?

* 449. Immunosuppressant therapy

Strongly Disagree 1	2	3	4	5	6	7	8	Strongly Agree 9
☆	☆	☆	☆	☆	☆	☆	☆	☆

* 450. Any additional considerations? E.g. dose, duration of use, specific medications over others?

* 451. Other medications not indicated above – please list

PATIENTS WITH IMMUNE DISORDERS

In patients with underlying immune disorders which symptoms would prompt you to test for NTM?

* 452. No symptoms

Strongly Disagree 1	2	3	4	5	6	7	8	Strongly Agree 9
★	★	★	★	★	★	★	★	★

* 453. Persistent cough

Strongly Disagree 1	2	3	4	5	6	7	8	Strongly Agree 9
★	★	★	★	★	★	★	★	★

* 454. Weight loss for no clear reason

Strongly Disagree 1	2	3	4	5	6	7	8	Strongly Agree 9
★	★	★	★	★	★	★	★	★

* 455. Night sweats

Strongly Disagree 1	2	3	4	5	6	7	8	Strongly Agree 9
★	★	★	★	★	★	★	★	★

* 456. Haemoptysis

Strongly Disagree 1	2	3	4	5	6	7	8	Strongly Agree 9
★	★	★	★	★	★	★	★	★

* 457. Increased or purulent sputum

Strongly Disagree 1	2	3	4	5	6	7	8	Strongly Agree 9
★	★	★	★	★	★	★	★	★

* 458. Persistent fatigue

Strongly Disagree 1	2	3	4	5	6	7	8	Strongly Agree 9

* 459. Increased frequency of exacerbations

Strongly Disagree 1	2	3	4	5	6	7	8	Strongly Agree 9

* 460. Fever

Strongly Disagree 1	2	3	4	5	6	7	8	Strongly Agree 9

* 461. Hoarseness

Strongly Disagree 1	2	3	4	5	6	7	8	Strongly Agree 9

* 462. GERD

Strongly Disagree 1	2	3	4	5	6	7	8	Strongly Agree 9

* 463. Decline in FEV1 of >20%

Strongly Disagree 1	2	3	4	5	6	7	8	Strongly Agree 9

* 464. Shortness of breath

Strongly Disagree 1	2	3	4	5	6	7	8	Strongly Agree 9

* 465. Combination of ≥2 or more symptoms outlined above

Strongly Disagree 1	2	3	4	5	6	7	8	Strongly Agree 9
★	★	★	★	★	★	★	★	★

* 466. Radiology suggestive of NTM

Strongly Disagree 1	2	3	4	5	6	7	8	Strongly Agree 9
★	★	★	★	★	★	★	★	★

PATIENTS WITH IMMUNE DISORDERS AND MEDICATIONS

Which of the following medications patients with immune disorders are taking would make you consider testing for NTM?

Patients in receipt of…

* 467. Macrolide therapy

Strongly Disagree 1	2	3	4	5	6	7	8	Strongly Agree 9
☆	☆	☆	☆	☆	☆	☆	☆	☆

* 468. Any additional considerations? E.g. dose, type of macrolide, duration of use…

* 469. Inhaled corticosteroids

Strongly Disagree 1	2	3	4	5	6	7	8	Strongly Agree 9
☆	☆	☆	☆	☆	☆	☆	☆	☆

* 470. Any additional considerations? E.g. dose, duration of use, specific steroids?

* 471. Oral corticosteroids

Strongly Disagree 1	2	3	4	5	6	7	8	Strongly Agree 9
☆	☆	☆	☆	☆	☆	☆	☆	☆

* 472. Any additional considerations? E.g. dose, duration of use, specific steroids?

* 473. Monoclonal antibodies

Strongly Disagree 1	2	3	4	5	6	7	8	Strongly Agree 9
☆	☆	☆	☆	☆	☆	☆	☆	☆

* 474. Any additional considerations? E.g. dose, duration of use, specific medications over others?

* 475. Biologic therapy e.g. immunomodulators

Strongly Disagree 1	2	3	4	5	6	7	8	Strongly Agree 9
☆	☆	☆	☆	☆	☆	☆	☆	☆

* 476. Any additional considerations? E.g. dose, duration of use, specific medications over others?

* 477. IV corticosteroids

Strongly Disagree 1	2	3	4	5	6	7	8	Strongly Agree 9
☆	☆	☆	☆	☆	☆	☆	☆	☆

* 478. Any additional considerations? E.g. dose, duration of use, specific steroids?

* 479. Immunosuppressant therapy

Strongly Disagree 1	2	3	4	5	6	7	8	Strongly Agree 9
☆	☆	☆	☆	☆	☆	☆	☆	☆

* 480. Any additional considerations? E.g. dose, duration of use, specific medications over others?

* 481. Other medications not indicated above – please list

PATIENTS WITH CONCOMITANT BACTERIAL INFECTIONS

In patients with concomitant bacterial infection which symptoms would prompt you to test for NTM?

* 482. No symptoms

Strongly Disagree 1	2	3	4	5	6	7	8	Strongly Agree 9
★	★	★	★	★	★	★	★	★

* 483. Persistent cough

Strongly Disagree 1	2	3	4	5	6	7	8	Strongly Agree 9
★	★	★	★	★	★	★	★	★

* 484. Weight loss for no clear reason

Strongly Disagree 1	2	3	4	5	6	7	8	Strongly Agree 9
★	★	★	★	★	★	★	★	★

* 485. Night sweats

Strongly Disagree 1	2	3	4	5	6	7	8	Strongly Agree 9
★	★	★	★	★	★	★	★	★

* 486. Haemoptysis

Strongly Disagree 1	2	3	4	5	6	7	8	Strongly Agree 9
★	★	★	★	★	★	★	★	★

* 487. Increased or purulent sputum

Strongly Disagree 1	2	3	4	5	6	7	8	Strongly Agree 9
★	★	★	★	★	★	★	★	★

* 488. Persistent fatigue

Strongly Disagree 1	2	3	4	5	6	7	8	Strongly Agree 9
★	★	★	★	★	★	★	★	★

* 489. Increased frequency of exacerbations

Strongly Disagree 1	2	3	4	5	6	7	8	Strongly Agree 9
★	★	★	★	★	★	★	★	★

* 490. Fever

Strongly Disagree 1	2	3	4	5	6	7	8	Strongly Agree 9
★	★	★	★	★	★	★	★	★

* 491. Hoarseness

Strongly Disagree 1	2	3	4	5	6	7	8	Strongly Agree 9
★	★	★	★	★	★	★	★	★

* 492. GERD

Strongly Disagree 1	2	3	4	5	6	7	8	Strongly Agree 9
★	★	★	★	★	★	★	★	★

* 493. Decline in FEV1 of >20%

Strongly Disagree 1	2	3	4	5	6	7	8	Strongly Agree 9
★	★	★	★	★	★	★	★	★

* 494. Shortness of breath

Strongly Disagree 1	2	3	4	5	6	7	8	Strongly Agree 9
★	★	★	★	★	★	★	★	★

* 495. Combination of ≥2 or more symptoms outlined above

Strongly Disagree 1	2	3	4	5	6	7	8	Strongly Agree 9
☆	☆	☆	☆	☆	☆	☆	☆	☆

* 496. Radiology suggestive of NTM

Strongly Disagree 1	2	3	4	5	6	7	8	Strongly Agree 9
☆	☆	☆	☆	☆	☆	☆	☆	☆

PATIENTS WITH CONCOMITANT BACTERIAL INFECTIONS AND MEDICATIONS

Which of the following medications patients with concomitant bacterial infections such as P. aeruginosa, S. aureus or others are taking would make you consider testing for NTM?

Patients in receipt of…

* 497. Macrolide therapy

Strongly Disagree 1	2	3	4	5	6	7	8	Strongly Agree 9
★	★	★	★	★	★	★	★	★

* 498. Any additional considerations? E.g. dose, type of macrolide, duration of use…

* 499. Inhaled corticosteroids

Strongly Disagree 1	2	3	4	5	6	7	8	Strongly Agree 9
★	★	★	★	★	★	★	★	★

* 500. Any additional considerations? E.g. dose, duration of use, specific steroids?

* 501. Oral corticosteroids

Strongly Disagree 1	2	3	4	5	6	7	8	Strongly Agree 9
★	★	★	★	★	★	★	★	★

* 502. Any additional considerations? E.g. dose, duration of use, specific steroids?

* 503. Monoclonal antibodies

Strongly Disagree 1	2	3	4	5	6	7	8	Strongly Agree 9
★	★	★	★	★	★	★	★	★

* 504. Any additional considerations? E.g. dose, duration of use, specific medications over others?

* 505. Biologic therapy e.g. immunomodulators

Strongly Disagree 1	2	3	4	5	6	7	8	Strongly Agree 9
★	★	★	★	★	★	★	★	★

* 506. Any additional considerations? E.g. dose, duration of use, specific medications over others?

* 507. IV corticosteroids

Strongly Disagree 1	2	3	4	5	6	7	8	Strongly Agree 9
★	★	★	★	★	★	★	★	★

* 508. Any additional considerations? E.g. dose, duration of use, specific steroids?

* 509. Immunosuppressant therapy

Strongly Disagree 1	2	3	4	5	6	7	8	Strongly Agree 9
★	★	★	★	★	★	★	★	★

* 510. Any additional considerations? E.g. dose, duration of use, specific medications over others?

* 511. Other medications not indicated above – please list

Delphi process – Round 2 Questionnaire

Section 1: NTM symptoms in possible at-risk patients

In this section questions explore symptoms for NTM-PD that were identified from the survey and the meta-analysis and explore when you might consider testing in patients with underlying comorbidities e.g. respiratory disease, other diseases, reductions in effective bodily functions as well as those in receipt of specific medications.

* 1. In order to facilitate discussion within the Delphi panel meetings, please add your name below. (Your identity is only visible to Highfield to facilitate effective development of the meeting agenda and discussion topics)

No respiratory disease

All adults with no diagnosed respiratory disease who have one or more of the following symptoms should be tested for NTM

* 2. No symptoms

Strongly disagree 1	2	3	4	5	6	7	8	Strongly Agree 9
☆	☆	☆	☆	☆	☆	☆	☆	☆

* 3. Persistent cough

Strongly Disagree 1	2	3	4	5	6	7	8	Strongly Agree 9
☆	☆	☆	☆	☆	☆	☆	☆	☆

* 4. Unexplained weight loss

Strongly Disagree 1	2	3	4	5	6	7	8	Strongly Agree 9
☆	☆	☆	☆	☆	☆	☆	☆	☆

* 5. Night sweats

Strongly Disagree 1	2	3	4	5	6	7	8	Strongly Agree 9
☆	☆	☆	☆	☆	☆	☆	☆	☆

* 6. Haemoptysis

Strongly Disagree 1	2	3	4	5	6	7	8	Strongly Agree 9
☆	☆	☆	☆	☆	☆	☆	☆	☆

* 7. Persistent sputum production

Strongly Disagree 1	2	3	4	5	6	7	8	Strongly Agree 9
☆	☆	☆	☆	☆	☆	☆	☆	☆

* 8. Persistent fatigue

Strongly Disagree 1	2	3	4	5	6	7	8	Strongly Agree 9
☆	☆	☆	☆	☆	☆	☆	☆	☆

* 9. Recurrent respiratory infection

Strongly Disagree 1	2	3	4	5	6	7	8	Strongly Agree 9
☆	☆	☆	☆	☆	☆	☆	☆	☆

* 10. Persistent unexplained fever

Strongly Disagree 1	2	3	4	5	6	7	8	Strongly Agree 9
☆	☆	☆	☆	☆	☆	☆	☆	☆

* 11. Persistent shortness of breath

Strongly Disagree 1	2	3	4	5	6	7	8	Strongly Agree 9
☆	☆	☆	☆	☆	☆	☆	☆	☆

* 12. Combination of ≥2 symptoms outlined above

Strongly Disagree 1	2	3	4	5	6	7	8	Strongly Agree 9
☆	☆	☆	☆	☆	☆	☆	☆	☆

* 13. Combination of ≥3 symptoms outlined above

Strongly Disagree 1	2	3	4	5	6	7	8	Strongly Agree 9
☆	☆	☆	☆	☆	☆	☆	☆	☆

* 14. Radiology suggestive of NTM

Strongly Disagree 1	2	3	4	5	6	7	8	Strongly Agree 9
☆	☆	☆	☆	☆	☆	☆	☆	☆

* 15. Before starting macrolides

Strongly Disagree 1	2	3	4	5	6	7	8	Strongly Agree 9
☆	☆	☆	☆	☆	☆	☆	☆	☆

* 16. In receipt of macrolides

Strongly Disagree 1	2	3	4	5	6	7	8	Strongly Agree 9
☆	☆	☆	☆	☆	☆	☆	☆	☆

* 17. In receipt of inhaled steroids

Strongly Disagree 1	2	3	4	5	6	7	8	Strongly Agree 9
☆	☆	☆	☆	☆	☆	☆	☆	☆

* 18. In receipt of immunosuppressive/immune disorder drugs

Strongly Disagree 1	2	3	4	5	6	7	8	Strongly Agree 9
☆	☆	☆	☆	☆	☆	☆	☆	☆

NCFBE

All adults with NCFBE who have one or more of the following symptoms should be tested for NTM

* 19. No symptoms

Strongly disagree 1	2	3	4	5	6	7	8	Strongly Agree 9
☆	☆	☆	☆	☆	☆	☆	☆	☆

* 20. Persistent cough

Strongly Disagree 1	2	3	4	5	6	7	8	Strongly Agree 9
☆	☆	☆	☆	☆	☆	☆	☆	☆

* 21. Unexplained weight loss

Strongly Disagree 1	2	3	4	5	6	7	8	Strongly Agree 9
☆	☆	☆	☆	☆	☆	☆	☆	☆

* 22. Night sweats

Strongly Disagree 1	2	3	4	5	6	7	8	Strongly Agree 9
☆	☆	☆	☆	☆	☆	☆	☆	☆

* 23. Haemoptysis

Strongly Disagree 1	2	3	4	5	6	7	8	Strongly Agree 9
☆	☆	☆	☆	☆	☆	☆	☆	☆

* 24. Persistent sputum production

Strongly Disagree 1	2	3	4	5	6	7	8	Strongly Agree 9
☆	☆	☆	☆	☆	☆	☆	☆	☆

* 25. Persistent fatigue

Strongly Disagree 1	2	3	4	5	6	7	8	Strongly Agree 9
☆	☆	☆	☆	☆	☆	☆	☆	☆

* 26. Recurrent respiratory infection

Strongly Disagree 1	2	3	4	5	6	7	8	Strongly Agree 9
☆	☆	☆	☆	☆	☆	☆	☆	☆

* 27. Persistent unexplained fever

Strongly Disagree 1	2	3	4	5	6	7	8	Strongly Agree 9
☆	☆	☆	☆	☆	☆	☆	☆	☆

* 28. Persistent shortness of breath

Strongly Disagree 1	2	3	4	5	6	7	8	Strongly Agree 9
☆	☆	☆	☆	☆	☆	☆	☆	☆

* 29. Combination of ≥2 symptoms outlined above

Strongly Disagree 1	2	3	4	5	6	7	8	Strongly Agree 9
☆	☆	☆	☆	☆	☆	☆	☆	☆

* 30. Combination of ≥3 symptoms outlined above

Strongly Disagree 1	2	3	4	5	6	7	8	Strongly Agree 9
☆	☆	☆	☆	☆	☆	☆	☆	☆

* 31. Radiology suggestive of NTM

Strongly Disagree 1	2	3	4	5	6	7	8	Strongly Agree 9
☆	☆	☆	☆	☆	☆	☆	☆	☆

* 32. Before starting macrolides

Strongly Disagree 1	2	3	4	5	6	7	8	Strongly Agree 9
★	★	★	★	★	★	★	★	★

* 33. In receipt of macrolides

Strongly Disagree 1	2	3	4	5	6	7	8	Strongly Agree 9
★	★	★	★	★	★	★	★	★

* 34. In receipt of inhaled steroids

Strongly Disagree 1	2	3	4	5	6	7	8	Strongly Agree 9
★	★	★	★	★	★	★	★	★

* 35. In receipt of immunosuppressive/immune disorder drugs

Strongly Disagree 1	2	3	4	5	6	7	8	Strongly Agree 9
★	★	★	★	★	★	★	★	★

Asthma

All adults with asthma who have one or more of the following symptoms should be tested for NTM

* 36. No symptoms

Strongly disagree 1	2	3	4	5	6	7	8	Strongly Agree 9
★	★	★	★	★	★	★	★	★

* 37. Persistent cough

Strongly Disagree 1	2	3	4	5	6	7	8	Strongly Agree 9
★	★	★	★	★	★	★	★	★

* 38. Unexplained weight loss

Strongly Disagree 1	2	3	4	5	6	7	8	Strongly Agree 9
★	★	★	★	★	★	★	★	★

* 39. Night sweats

Strongly Disagree 1	2	3	4	5	6	7	8	Strongly Agree 9
★	★	★	★	★	★	★	★	★

* 40. Haemoptysis

Strongly Disagree 1	2	3	4	5	6	7	8	Strongly Agree 9
★	★	★	★	★	★	★	★	★

* 41. Persistent sputum production

Strongly Disagree 1	2	3	4	5	6	7	8	Strongly Agree 9
★	★	★	★	★	★	★	★	★

* 42. Persistent fatigue

Strongly Disagree 1	2	3	4	5	6	7	8	Strongly Agree 9
☆	☆	☆	☆	☆	☆	☆	☆	☆

* 43. Recurrent respiratory infection

Strongly Disagree 1	2	3	4	5	6	7	8	Strongly Agree 9
☆	☆	☆	☆	☆	☆	☆	☆	☆

* 44. Persistent unexplained fever

Strongly Disagree 1	2	3	4	5	6	7	8	Strongly Agree 9
☆	☆	☆	☆	☆	☆	☆	☆	☆

* 45. Persistent shortness of breath

Strongly Disagree 1	2	3	4	5	6	7	8	Strongly Agree 9
☆	☆	☆	☆	☆	☆	☆	☆	☆

* 46. Combination of ≥2 symptoms outlined above

Strongly Disagree 1	2	3	4	5	6	7	8	Strongly Agree 9
☆	☆	☆	☆	☆	☆	☆	☆	☆

* 47. Combination of ≥3 symptoms outlined above

Strongly Disagree 1	2	3	4	5	6	7	8	Strongly Agree 9
☆	☆	☆	☆	☆	☆	☆	☆	☆

* 48. Radiology suggestive of NTM

Strongly Disagree 1	2	3	4	5	6	7	8	Strongly Agree 9
☆	☆	☆	☆	☆	☆	☆	☆	☆

* 49. Before starting macrolides

Strongly Disagree 1	2	3	4	5	6	7	8	Strongly Agree 9
★	★	★	★	★	★	★	★	★

* 50. In receipt of macrolides

Strongly Disagree 1	2	3	4	5	6	7	8	Strongly Agree 9
★	★	★	★	★	★	★	★	★

* 51. In receipt of inhaled steroids

Strongly Disagree 1	2	3	4	5	6	7	8	Strongly Agree 9
★	★	★	★	★	★	★	★	★

* 52. In receipt of immunosuppressive/immune disorder drugs

Strongly Disagree 1	2	3	4	5	6	7	8	Strongly Agree 9
★	★	★	★	★	★	★	★	★

CF

All adults with CF who have one or more of the following symptoms should be tested for NTM

* 53. No symptoms

Strongly disagree 1	2	3	4	5	6	7	8	Strongly Agree 9
★	★	★	★	★	★	★	★	★

* 54. Persistent cough

Strongly Disagree 1	2	3	4	5	6	7	8	Strongly Agree 9
★	★	★	★	★	★	★	★	★

* 55. Unexplained weight loss

Strongly Disagree 1	2	3	4	5	6	7	8	Strongly Agree 9
★	★	★	★	★	★	★	★	★

* 56. Night sweats

Strongly Disagree 1	2	3	4	5	6	7	8	Strongly Agree 9
★	★	★	★	★	★	★	★	★

* 57. Haemoptysis

Strongly Disagree 1	2	3	4	5	6	7	8	Strongly Agree 9
★	★	★	★	★	★	★	★	★

* 58. Persistent sputum production

Strongly Disagree 1	2	3	4	5	6	7	8	Strongly Agree 9
★	★	★	★	★	★	★	★	★

* 59. Persistent fatigue

Strongly Disagree 1	2	3	4	5	6	7	8	Strongly Agree 9
☆	☆	☆	☆	☆	☆	☆	☆	☆

* 60. Recurrent respiratory infection

Strongly Disagree 1	2	3	4	5	6	7	8	Strongly Agree 9
☆	☆	☆	☆	☆	☆	☆	☆	☆

* 61. Persistent unexplained fever

Strongly Disagree 1	2	3	4	5	6	7	8	Strongly Agree 9
☆	☆	☆	☆	☆	☆	☆	☆	☆

* 62. Persistent shortness of breath

Strongly Disagree 1	2	3	4	5	6	7	8	Strongly Agree 9
☆	☆	☆	☆	☆	☆	☆	☆	☆

* 63. Combination of ≥2 symptoms outlined above

Strongly Disagree 1	2	3	4	5	6	7	8	Strongly Agree 9
☆	☆	☆	☆	☆	☆	☆	☆	☆

* 64. Combination of ≥3 symptoms outlined above

Strongly Disagree 1	2	3	4	5	6	7	8	Strongly Agree 9
☆	☆	☆	☆	☆	☆	☆	☆	☆

* 65. Radiology suggestive of NTM

Strongly Disagree 1	2	3	4	5	6	7	8	Strongly Agree 9
☆	☆	☆	☆	☆	☆	☆	☆	☆

* 66. Before starting macrolides

Strongly Disagree 1	2	3	4	5	6	7	8	Strongly Agree 9
☆	☆	☆	☆	☆	☆	☆	☆	☆

* 67. In receipt of macrolides

Strongly Disagree 1	2	3	4	5	6	7	8	Strongly Agree 9
☆	☆	☆	☆	☆	☆	☆	☆	☆

* 68. In receipt of inhaled steroids

Strongly Disagree 1	2	3	4	5	6	7	8	Strongly Agree 9
☆	☆	☆	☆	☆	☆	☆	☆	☆

* 69. In receipt of immunosuppressive/immune disorder drugs

Strongly Disagree 1	2	3	4	5	6	7	8	Strongly Agree 9
☆	☆	☆	☆	☆	☆	☆	☆	☆

COPD

All adults with COPD who have one or more of the following symptoms should be tested for NTM

* 70. No symptoms

Strongly disagree 1	2	3	4	5	6	7	8	Strongly Agree 9
☆	☆	☆	☆	☆	☆	☆	☆	☆

* 71. Persistent cough

Strongly Disagree 1	2	3	4	5	6	7	8	Strongly Agree 9
☆	☆	☆	☆	☆	☆	☆	☆	☆

* 72. Unexplained weight loss

Strongly Disagree 1	2	3	4	5	6	7	8	Strongly Agree 9
☆	☆	☆	☆	☆	☆	☆	☆	☆

* 73. Night sweats

Strongly Disagree 1	2	3	4	5	6	7	8	Strongly Agree 9
☆	☆	☆	☆	☆	☆	☆	☆	☆

* 74. Haemoptysis

Strongly Disagree 1	2	3	4	5	6	7	8	Strongly Agree 9
☆	☆	☆	☆	☆	☆	☆	☆	☆

* 75. Persistent sputum production

Strongly Disagree 1	2	3	4	5	6	7	8	Strongly Agree 9
☆	☆	☆	☆	☆	☆	☆	☆	☆

* 76. Persistent fatigue

Strongly Disagree 1	2	3	4	5	6	7	8	Strongly Agree 9
☆	☆	☆	☆	☆	☆	☆	☆	☆

* 77. Recurrent respiratory infection

Strongly Disagree 1	2	3	4	5	6	7	8	Strongly Agree 9
☆	☆	☆	☆	☆	☆	☆	☆	☆

* 78. Persistent unexplained fever

Strongly Disagree 1	2	3	4	5	6	7	8	Strongly Agree 9
☆	☆	☆	☆	☆	☆	☆	☆	☆

* 79. Persistent shortness of breath

Strongly Disagree 1	2	3	4	5	6	7	8	Strongly Agree 9
☆	☆	☆	☆	☆	☆	☆	☆	☆

* 80. Combination of ≥2 symptoms outlined above

Strongly Disagree 1	2	3	4	5	6	7	8	Strongly Agree 9
☆	☆	☆	☆	☆	☆	☆	☆	☆

* 81. Combination of ≥3 symptoms outlined above

Strongly Disagree 1	2	3	4	5	6	7	8	Strongly Agree 9
☆	☆	☆	☆	☆	☆	☆	☆	☆

* 82. Radiology suggestive of NTM

Strongly Disagree 1	2	3	4	5	6	7	8	Strongly Agree 9
☆	☆	☆	☆	☆	☆	☆	☆	☆

* 83. Before starting macrolides

Strongly Disagree 1	2	3	4	5	6	7	8	Strongly Agree 9
★	★	★	★	★	★	★	★	★

* 84. In receipt of macrolides

Strongly Disagree 1	2	3	4	5	6	7	8	Strongly Agree 9
★	★	★	★	★	★	★	★	★

* 85. In receipt of inhaled steroids

Strongly Disagree 1	2	3	4	5	6	7	8	Strongly Agree 9
★	★	★	★	★	★	★	★	★

* 86. In receipt of immunosuppressive/immune disorder drugs

Strongly Disagree 1	2	3	4	5	6	7	8	Strongly Agree 9
★	★	★	★	★	★	★	★	★

Previous history of TB

All adults with a previous history of TB who have one or more of the following symptoms should be tested for NTM

* 87. No symptoms

Strongly disagree 1	2	3	4	5	6	7	8	Strongly Agree 9
☆	☆	☆	☆	☆	☆	☆	☆	☆

* 88. Persistent cough

Strongly Disagree 1	2	3	4	5	6	7	8	Strongly Agree 9
☆	☆	☆	☆	☆	☆	☆	☆	☆

* 89. Unexplained weight loss

Strongly Disagree 1	2	3	4	5	6	7	8	Strongly Agree 9
☆	☆	☆	☆	☆	☆	☆	☆	☆

* 90. Night sweats

Strongly Disagree 1	2	3	4	5	6	7	8	Strongly Agree 9
☆	☆	☆	☆	☆	☆	☆	☆	☆

* 91. Haemoptysis

Strongly Disagree 1	2	3	4	5	6	7	8	Strongly Agree 9
☆	☆	☆	☆	☆	☆	☆	☆	☆

* 92. Persistent sputum production

Strongly Disagree 1	2	3	4	5	6	7	8	Strongly Agree 9
☆	☆	☆	☆	☆	☆	☆	☆	☆

* 93. Persistent fatigue

Strongly Disagree 1	2	3	4	5	6	7	8	Strongly Agree 9
☆	☆	☆	☆	☆	☆	☆	☆	☆

* 94. Recurrent respiratory infection

Strongly Disagree 1	2	3	4	5	6	7	8	Strongly Agree 9
☆	☆	☆	☆	☆	☆	☆	☆	☆

* 95. Persistent unexplained fever

Strongly Disagree 1	2	3	4	5	6	7	8	Strongly Agree 9
☆	☆	☆	☆	☆	☆	☆	☆	☆

* 96. Persistent shortness of breath

Strongly Disagree 1	2	3	4	5	6	7	8	Strongly Agree 9
☆	☆	☆	☆	☆	☆	☆	☆	☆

* 97. Combination of ≥2 symptoms outlined above

Strongly Disagree 1	2	3	4	5	6	7	8	Strongly Agree 9
☆	☆	☆	☆	☆	☆	☆	☆	☆

* 98. Combination of ≥3 symptoms outlined above

Strongly Disagree 1	2	3	4	5	6	7	8	Strongly Agree 9
☆	☆	☆	☆	☆	☆	☆	☆	☆

* 99. Radiology suggestive of NTM

Strongly Disagree 1	2	3	4	5	6	7	8	Strongly Agree 9
☆	☆	☆	☆	☆	☆	☆	☆	☆

* 100. Before starting macrolides

Strongly Disagree 1	2	3	4	5	6	7	8	Strongly Agree 9
☆	☆	☆	☆	☆	☆	☆	☆	☆

* 101. In receipt of macrolides

Strongly Disagree 1	2	3	4	5	6	7	8	Strongly Agree 9
☆	☆	☆	☆	☆	☆	☆	☆	☆

* 102. In receipt of inhaled steroids

Strongly Disagree 1	2	3	4	5	6	7	8	Strongly Agree 9
☆	☆	☆	☆	☆	☆	☆	☆	☆

* 103. In receipt of immunosuppressive/immune disorder drugs

Strongly Disagree 1	2	3	4	5	6	7	8	Strongly Agree 9
☆	☆	☆	☆	☆	☆	☆	☆	☆

ILD

All adults with ILD who have one or more of the following symptoms should be tested for NTM

* 104. No symptoms

Strongly disagree 1	2	3	4	5	6	7	8	Strongly Agree 9
★	★	★	★	★	★	★	★	★

* 105. Persistent cough

Strongly Disagree 1	2	3	4	5	6	7	8	Strongly Agree 9
★	★	★	★	★	★	★	★	★

* 106. Unexplained weight loss

Strongly Disagree 1	2	3	4	5	6	7	8	Strongly Agree 9
★	★	★	★	★	★	★	★	★

* 107. Night sweats

Strongly Disagree 1	2	3	4	5	6	7	8	Strongly Agree 9
★	★	★	★	★	★	★	★	★

* 108. Haemoptysis

Strongly Disagree 1	2	3	4	5	6	7	8	Strongly Agree 9
★	★	★	★	★	★	★	★	★

* 109. Persistent sputum production

Strongly Disagree 1	2	3	4	5	6	7	8	Strongly Agree 9
★	★	★	★	★	★	★	★	★

* 110. Persistent fatigue

Strongly Disagree 1	2	3	4	5	6	7	8	Strongly Agree 9
☆	☆	☆	☆	☆	☆	☆	☆	☆

* 111. Recurrent respiratory infection

Strongly Disagree 1	2	3	4	5	6	7	8	Strongly Agree 9
☆	☆	☆	☆	☆	☆	☆	☆	☆

* 112. Persistent unexplained fever

Strongly Disagree 1	2	3	4	5	6	7	8	Strongly Agree 9
☆	☆	☆	☆	☆	☆	☆	☆	☆

* 113. Persistent shortness of breath

Strongly Disagree 1	2	3	4	5	6	7	8	Strongly Agree 9
☆	☆	☆	☆	☆	☆	☆	☆	☆

* 114. Combination of ≥2 symptoms outlined above

Strongly Disagree 1	2	3	4	5	6	7	8	Strongly Agree 9
☆	☆	☆	☆	☆	☆	☆	☆	☆

* 115. Combination of ≥3 symptoms outlined above

Strongly Disagree 1	2	3	4	5	6	7	8	Strongly Agree 9
☆	☆	☆	☆	☆	☆	☆	☆	☆

* 116. Radiology suggestive of NTM

Strongly Disagree 1	2	3	4	5	6	7	8	Strongly Agree 9
☆	☆	☆	☆	☆	☆	☆	☆	☆

* 117. Before starting macrolides

Strongly Disagree 1	2	3	4	5	6	7	8	Strongly Agree 9
☆	☆	☆	☆	☆	☆	☆	☆	☆

* 118. In receipt of macrolides

Strongly Disagree 1	2	3	4	5	6	7	8	Strongly Agree 9
☆	☆	☆	☆	☆	☆	☆	☆	☆

* 119. In receipt of inhaled steroids

Strongly Disagree 1	2	3	4	5	6	7	8	Strongly Agree 9
☆	☆	☆	☆	☆	☆	☆	☆	☆

* 120. In receipt of immunosuppressive/immune disorder drugs

Strongly Disagree 1	2	3	4	5	6	7	8	Strongly Agree 9
☆	☆	☆	☆	☆	☆	☆	☆	☆

Fungal lung disease

All adults with fungal lung disease who have one or more of the following symptoms should be tested for NTM

* 121. No symptoms

Strongly disagree 1	2	3	4	5	6	7	8	Strongly Agree 9
★	★	★	★	★	★	★	★	★

* 122. Persistent cough

Strongly Disagree 1	2	3	4	5	6	7	8	Strongly Agree 9
★	★	★	★	★	★	★	★	★

* 123. Unexplained weight loss

Strongly Disagree 1	2	3	4	5	6	7	8	Strongly Agree 9
★	★	★	★	★	★	★	★	★

* 124. Night sweats

Strongly Disagree 1	2	3	4	5	6	7	8	Strongly Agree 9
★	★	★	★	★	★	★	★	★

* 125. Haemoptysis

Strongly Disagree 1	2	3	4	5	6	7	8	Strongly Agree 9
★	★	★	★	★	★	★	★	★

* 126. Persistent sputum production

Strongly Disagree 1	2	3	4	5	6	7	8	Strongly Agree 9
★	★	★	★	★	★	★	★	★

* 127. Persistent fatigue

Strongly Disagree 1	2	3	4	5	6	7	8	Strongly Agree 9
☆	☆	☆	☆	☆	☆	☆	☆	☆

* 128. Recurrent respiratory infection

Strongly Disagree 1	2	3	4	5	6	7	8	Strongly Agree 9
☆	☆	☆	☆	☆	☆	☆	☆	☆

* 129. Persistent unexplained fever

Strongly Disagree 1	2	3	4	5	6	7	8	Strongly Agree 9
☆	☆	☆	☆	☆	☆	☆	☆	☆

* 130. Persistent shortness of breath

Strongly Disagree 1	2	3	4	5	6	7	8	Strongly Agree 9
☆	☆	☆	☆	☆	☆	☆	☆	☆

* 131. Combination of ≥2 symptoms outlined above

Strongly Disagree 1	2	3	4	5	6	7	8	Strongly Agree 9
☆	☆	☆	☆	☆	☆	☆	☆	☆

* 132. Combination of ≥3 symptoms outlined above

Strongly Disagree 1	2	3	4	5	6	7	8	Strongly Agree 9
☆	☆	☆	☆	☆	☆	☆	☆	☆

* 133. Radiology suggestive of NTM

Strongly Disagree 1	2	3	4	5	6	7	8	Strongly Agree 9
☆	☆	☆	☆	☆	☆	☆	☆	☆

* 134. Before starting macrolides

Strongly Disagree 1	2	3	4	5	6	7	8	Strongly Agree 9
★	★	★	★	★	★	★	★	★

* 135. In receipt of macrolides

Strongly Disagree 1	2	3	4	5	6	7	8	Strongly Agree 9
★	★	★	★	★	★	★	★	★

* 136. In receipt of inhaled steroids

Strongly Disagree 1	2	3	4	5	6	7	8	Strongly Agree 9
★	★	★	★	★	★	★	★	★

* 137. In receipt of immunosuppressive/immune disorder drugs

Strongly Disagree 1	2	3	4	5	6	7	8	Strongly Agree 9
★	★	★	★	★	★	★	★	★

Immunosuppressed patients

All adults with immunosuppression who have one or more of the following symptoms should be tested for NTM

* 138. No symptoms

Strongly disagree 1	2	3	4	5	6	7	8	Strongly Agree 9
☆	☆	☆	☆	☆	☆	☆	☆	☆

* 139. Persistent cough

Strongly Disagree 1	2	3	4	5	6	7	8	Strongly Agree 9
☆	☆	☆	☆	☆	☆	☆	☆	☆

* 140. Unexplained weight loss

Strongly Disagree 1	2	3	4	5	6	7	8	Strongly Agree 9
☆	☆	☆	☆	☆	☆	☆	☆	☆

* 141. Night sweats

Strongly Disagree 1	2	3	4	5	6	7	8	Strongly Agree 9
☆	☆	☆	☆	☆	☆	☆	☆	☆

* 142. Haemoptysis

Strongly Disagree 1	2	3	4	5	6	7	8	Strongly Agree 9
☆	☆	☆	☆	☆	☆	☆	☆	☆

* 143. Persistent sputum production

Strongly Disagree 1	2	3	4	5	6	7	8	Strongly Agree 9
☆	☆	☆	☆	☆	☆	☆	☆	☆

* 144. Persistent fatigue

Strongly Disagree 1	2	3	4	5	6	7	8	Strongly Agree 9
☆	☆	☆	☆	☆	☆	☆	☆	☆

* 145. Recurrent respiratory infection

Strongly Disagree 1	2	3	4	5	6	7	8	Strongly Agree 9
☆	☆	☆	☆	☆	☆	☆	☆	☆

* 146. Persistent unexplained fever

Strongly Disagree 1	2	3	4	5	6	7	8	Strongly Agree 9
☆	☆	☆	☆	☆	☆	☆	☆	☆

* 147. Persistent shortness of breath

Strongly Disagree 1	2	3	4	5	6	7	8	Strongly Agree 9
☆	☆	☆	☆	☆	☆	☆	☆	☆

* 148. Combination of ≥2 symptoms outlined above

Strongly Disagree 1	2	3	4	5	6	7	8	Strongly Agree 9
☆	☆	☆	☆	☆	☆	☆	☆	☆

* 149. Combination of ≥3 symptoms outlined above

Strongly Disagree 1	2	3	4	5	6	7	8	Strongly Agree 9
☆	☆	☆	☆	☆	☆	☆	☆	☆

* 150. Radiology suggestive of NTM

Strongly Disagree 1	2	3	4	5	6	7	8	Strongly Agree 9
☆	☆	☆	☆	☆	☆	☆	☆	☆

* 151. Before starting macrolides

Strongly Disagree 1	2	3	4	5	6	7	8	Strongly Agree 9
★	★	★	★	★	★	★	★	★

* 152. In receipt of macrolides

Strongly Disagree 1	2	3	4	5	6	7	8	Strongly Agree 9
★	★	★	★	★	★	★	★	★

* 153. In receipt of inhaled steroids

Strongly Disagree 1	2	3	4	5	6	7	8	Strongly Agree 9
★	★	★	★	★	★	★	★	★

* 154. In receipt of immunosuppressive/immune disorder drugs

Strongly Disagree 1	2	3	4	5	6	7	8	Strongly Agree 9
★	★	★	★	★	★	★	★	★

Post-transplant patients

All adults post-transplant who have one or more of the following symptoms should be tested for NTM

* 155. No symptoms

Strongly disagree 1	2	3	4	5	6	7	8	Strongly Agree 9
★	★	★	★	★	★	★	★	★

* 156. Persistent cough

Strongly Disagree 1	2	3	4	5	6	7	8	Strongly Agree 9
★	★	★	★	★	★	★	★	★

* 157. Unexplained weight loss

Strongly Disagree 1	2	3	4	5	6	7	8	Strongly Agree 9
★	★	★	★	★	★	★	★	★

* 158. Night sweats

Strongly Disagree 1	2	3	4	5	6	7	8	Strongly Agree 9
★	★	★	★	★	★	★	★	★

* 159. Haemoptysis

Strongly Disagree 1	2	3	4	5	6	7	8	Strongly Agree 9
★	★	★	★	★	★	★	★	★

* 160. Persistent sputum production

Strongly Disagree 1	2	3	4	5	6	7	8	Strongly Agree 9
★	★	★	★	★	★	★	★	★

* 161. Persistent fatigue

Strongly Disagree 1	2	3	4	5	6	7	8	Strongly Agree 9
★	★	★	★	★	★	★	★	★

* 162. Recurrent respiratory infection

Strongly Disagree 1	2	3	4	5	6	7	8	Strongly Agree 9
★	★	★	★	★	★	★	★	★

* 163. Persistent unexplained fever

Strongly Disagree 1	2	3	4	5	6	7	8	Strongly Agree 9
★	★	★	★	★	★	★	★	★

* 164. Persistent shortness of breath

Strongly Disagree 1	2	3	4	5	6	7	8	Strongly Agree 9
★	★	★	★	★	★	★	★	★

* 165. Combination of ≥2 symptoms outlined above

Strongly Disagree 1	2	3	4	5	6	7	8	Strongly Agree 9
★	★	★	★	★	★	★	★	★

* 166. Combination of ≥3 symptoms outlined above

Strongly Disagree 1	2	3	4	5	6	7	8	Strongly Agree 9
★	★	★	★	★	★	★	★	★

* 167. Radiology suggestive of NTM

Strongly Disagree 1	2	3	4	5	6	7	8	Strongly Agree 9
★	★	★	★	★	★	★	★	★

* 168. Before starting macrolides

Strongly Disagree 1	2	3	4	5	6	7	8	Strongly Agree 9
★	★	★	★	★	★	★	★	★

* 169. In receipt of macrolides

Strongly Disagree 1	2	3	4	5	6	7	8	Strongly Agree 9
★	★	★	★	★	★	★	★	★

* 170. In receipt of inhaled steroids

Strongly Disagree 1	2	3	4	5	6	7	8	Strongly Agree 9
★	★	★	★	★	★	★	★	★

* 171. In receipt of immunosuppressive/immune disorder drugs

Strongly Disagree 1	2	3	4	5	6	7	8	Strongly Agree 9
★	★	★	★	★	★	★	★	★

Autoimmune patients

All adults with autoimmune disorders who have one or more of the following symptoms should be tested for NTM

* 172. No symptoms

Strongly disagree 1	2	3	4	5	6	7	8	Strongly Agree 9
☆	☆	☆	☆	☆	☆	☆	☆	☆

* 173. Persistent cough

Strongly Disagree 1	2	3	4	5	6	7	8	Strongly Agree 9
☆	☆	☆	☆	☆	☆	☆	☆	☆

* 174. Unexplained weight loss

Strongly Disagree 1	2	3	4	5	6	7	8	Strongly Agree 9
☆	☆	☆	☆	☆	☆	☆	☆	☆

* 175. Night sweats

Strongly Disagree 1	2	3	4	5	6	7	8	Strongly Agree 9
☆	☆	☆	☆	☆	☆	☆	☆	☆

* 176. Haemoptysis

Strongly Disagree 1	2	3	4	5	6	7	8	Strongly Agree 9
☆	☆	☆	☆	☆	☆	☆	☆	☆

* 177. Persistent sputum production

Strongly Disagree 1	2	3	4	5	6	7	8	Strongly Agree 9
☆	☆	☆	☆	☆	☆	☆	☆	☆

* 178. Persistent fatigue

Strongly Disagree 1	2	3	4	5	6	7	8	Strongly Agree 9
☆	☆	☆	☆	☆	☆	☆	☆	☆

* 179. Recurrent respiratory infection

Strongly Disagree 1	2	3	4	5	6	7	8	Strongly Agree 9
☆	☆	☆	☆	☆	☆	☆	☆	☆

* 180. Persistent unexplained fever

Strongly Disagree 1	2	3	4	5	6	7	8	Strongly Agree 9
☆	☆	☆	☆	☆	☆	☆	☆	☆

* 181. Persistent shortness of breath

Strongly Disagree 1	2	3	4	5	6	7	8	Strongly Agree 9
☆	☆	☆	☆	☆	☆	☆	☆	☆

* 182. Combination of ≥2 symptoms outlined above

Strongly Disagree 1	2	3	4	5	6	7	8	Strongly Agree 9
☆	☆	☆	☆	☆	☆	☆	☆	☆

* 183. Combination of ≥3 symptoms outlined above

Strongly Disagree 1	2	3	4	5	6	7	8	Strongly Agree 9
☆	☆	☆	☆	☆	☆	☆	☆	☆

* 184. Radiology suggestive of NTM

Strongly Disagree 1	2	3	4	5	6	7	8	Strongly Agree 9
☆	☆	☆	☆	☆	☆	☆	☆	☆

* 185. Before starting macrolides

Strongly Disagree 1	2	3	4	5	6	7	8	Strongly Agree 9
★	★	★	★	★	★	★	★	★

* 186. In receipt of macrolides

Strongly Disagree 1	2	3	4	5	6	7	8	Strongly Agree 9
★	★	★	★	★	★	★	★	★

* 187. In receipt of inhaled steroids

Strongly Disagree 1	2	3	4	5	6	7	8	Strongly Agree 9
★	★	★	★	★	★	★	★	★

* 188. In receipt of immunosuppressive/immune disorder drugs

Strongly Disagree 1	2	3	4	5	6	7	8	Strongly Agree 9
★	★	★	★	★	★	★	★	★

RA

All adults with RA who have one or more of the following symptoms should be tested for NTM

* 189. No symptoms

Strongly disagree 1	2	3	4	5	6	7	8	Strongly Agree 9
★	★	★	★	★	★	★	★	★

* 190. Persistent cough

Strongly Disagree 1	2	3	4	5	6	7	8	Strongly Agree 9
★	★	★	★	★	★	★	★	★

* 191. Unexplained weight loss

Strongly Disagree 1	2	3	4	5	6	7	8	Strongly Agree 9
★	★	★	★	★	★	★	★	★

* 192. Night sweats

Strongly Disagree 1	2	3	4	5	6	7	8	Strongly Agree 9
★	★	★	★	★	★	★	★	★

* 193. Haemoptysis

Strongly Disagree 1	2	3	4	5	6	7	8	Strongly Agree 9
★	★	★	★	★	★	★	★	★

* 194. Persistent sputum production

Strongly Disagree 1	2	3	4	5	6	7	8	Strongly Agree 9
★	★	★	★	★	★	★	★	★

* 195. Persistent fatigue

Strongly Disagree 1	2	3	4	5	6	7	8	Strongly Agree 9
★	★	★	★	★	★	★	★	★

* 196. Recurrent respiratory infection

Strongly Disagree 1	2	3	4	5	6	7	8	Strongly Agree 9
★	★	★	★	★	★	★	★	★

* 197. Persistent unexplained fever

Strongly Disagree 1	2	3	4	5	6	7	8	Strongly Agree 9
★	★	★	★	★	★	★	★	★

* 198. Persistent shortness of breath

Strongly Disagree 1	2	3	4	5	6	7	8	Strongly Agree 9
★	★	★	★	★	★	★	★	★

* 199. Combination of ≥2 symptoms outlined above

Strongly Disagree 1	2	3	4	5	6	7	8	Strongly Agree 9
★	★	★	★	★	★	★	★	★

* 200. Combination of ≥3 symptoms outlined above

Strongly Disagree 1	2	3	4	5	6	7	8	Strongly Agree 9
★	★	★	★	★	★	★	★	★

* 201. Radiology suggestive of NTM

Strongly Disagree 1	2	3	4	5	6	7	8	Strongly Agree 9
★	★	★	★	★	★	★	★	★

* 202. Before starting macrolides

Strongly Disagree 1	2	3	4	5	6	7	8	Strongly Agree 9
☆	☆	☆	☆	☆	☆	☆	☆	☆

* 203. In receipt of macrolides

Strongly Disagree 1	2	3	4	5	6	7	8	Strongly Agree 9
☆	☆	☆	☆	☆	☆	☆	☆	☆

* 204. In receipt of inhaled steroids

Strongly Disagree 1	2	3	4	5	6	7	8	Strongly Agree 9
☆	☆	☆	☆	☆	☆	☆	☆	☆

* 205. In receipt of immunosuppressive/immune disorder drugs

Strongly Disagree 1	2	3	4	5	6	7	8	Strongly Agree 9
☆	☆	☆	☆	☆	☆	☆	☆	☆

Renal disease

All adults with renal disease who have one or more of the following symptoms should be tested for NTM

* 206. No symptoms

Strongly disagree 1	2	3	4	5	6	7	8	Strongly Agree 9
★	★	★	★	★	★	★	★	★

* 207. Persistent cough

Strongly Disagree 1	2	3	4	5	6	7	8	Strongly Agree 9
★	★	★	★	★	★	★	★	★

* 208. Unexplained weight loss

Strongly Disagree 1	2	3	4	5	6	7	8	Strongly Agree 9
★	★	★	★	★	★	★	★	★

* 209. Night sweats

Strongly Disagree 1	2	3	4	5	6	7	8	Strongly Agree 9
★	★	★	★	★	★	★	★	★

* 210. Haemoptysis

Strongly Disagree 1	2	3	4	5	6	7	8	Strongly Agree 9
★	★	★	★	★	★	★	★	★

* 211. Persistent sputum production

Strongly Disagree 1	2	3	4	5	6	7	8	Strongly Agree 9
★	★	★	★	★	★	★	★	★

* 212. Persistent fatigue

Strongly Disagree 1	2	3	4	5	6	7	8	Strongly Agree 9
☆	☆	☆	☆	☆	☆	☆	☆	☆

* 213. Recurrent respiratory infection

Strongly Disagree 1	2	3	4	5	6	7	8	Strongly Agree 9
☆	☆	☆	☆	☆	☆	☆	☆	☆

* 214. Persistent unexplained fever

Strongly Disagree 1	2	3	4	5	6	7	8	Strongly Agree 9
☆	☆	☆	☆	☆	☆	☆	☆	☆

* 215. Persistent shortness of breath

Strongly Disagree 1	2	3	4	5	6	7	8	Strongly Agree 9
☆	☆	☆	☆	☆	☆	☆	☆	☆

* 216. Combination of ≥2 symptoms outlined above

Strongly Disagree 1	2	3	4	5	6	7	8	Strongly Agree 9
☆	☆	☆	☆	☆	☆	☆	☆	☆

* 217. Combination of ≥3 symptoms outlined above

Strongly Disagree 1	2	3	4	5	6	7	8	Strongly Agree 9
☆	☆	☆	☆	☆	☆	☆	☆	☆

* 218. Radiology suggestive of NTM

Strongly Disagree 1	2	3	4	5	6	7	8	Strongly Agree 9
☆	☆	☆	☆	☆	☆	☆	☆	☆

* 219. Before starting macrolides

Strongly Disagree 1	2	3	4	5	6	7	8	Strongly Agree 9
☆	☆	☆	☆	☆	☆	☆	☆	☆

* 220. In receipt of macrolides

Strongly Disagree 1	2	3	4	5	6	7	8	Strongly Agree 9
☆	☆	☆	☆	☆	☆	☆	☆	☆

* 221. In receipt of inhaled steroids

Strongly Disagree 1	2	3	4	5	6	7	8	Strongly Agree 9
☆	☆	☆	☆	☆	☆	☆	☆	☆

* 222. In receipt of immunosuppressive/immune disorder drugs

Strongly Disagree 1	2	3	4	5	6	7	8	Strongly Agree 9
☆	☆	☆	☆	☆	☆	☆	☆	☆

Diabetes

All adults with diabetes who have one or more of the following symptoms should be tested for NTM

* 223. No symptoms

Strongly disagree 1	2	3	4	5	6	7	8	Strongly Agree 9
★	★	★	★	★	★	★	★	★

* 224. Persistent cough

Strongly Disagree 1	2	3	4	5	6	7	8	Strongly Agree 9
★	★	★	★	★	★	★	★	★

* 225. Unexplained weight loss

Strongly Disagree 1	2	3	4	5	6	7	8	Strongly Agree 9
★	★	★	★	★	★	★	★	★

* 226. Night sweats

Strongly Disagree 1	2	3	4	5	6	7	8	Strongly Agree 9
★	★	★	★	★	★	★	★	★

* 227. Haemoptysis

Strongly Disagree 1	2	3	4	5	6	7	8	Strongly Agree 9
★	★	★	★	★	★	★	★	★

* 228. Persistent sputum production

Strongly Disagree 1	2	3	4	5	6	7	8	Strongly Agree 9
★	★	★	★	★	★	★	★	★

* 229. Persistent fatigue

Strongly Disagree 1	2	3	4	5	6	7	8	Strongly Agree 9
★	★	★	★	★	★	★	★	★

* 230. Recurrent respiratory infection

Strongly Disagree 1	2	3	4	5	6	7	8	Strongly Agree 9
★	★	★	★	★	★	★	★	★

* 231. Persistent unexplained fever

Strongly Disagree 1	2	3	4	5	6	7	8	Strongly Agree 9
★	★	★	★	★	★	★	★	★

* 232. Persistent shortness of breath

Strongly Disagree 1	2	3	4	5	6	7	8	Strongly Agree 9
★	★	★	★	★	★	★	★	★

* 233. Combination of ≥2 symptoms outlined above

Strongly Disagree 1	2	3	4	5	6	7	8	Strongly Agree 9
★	★	★	★	★	★	★	★	★

* 234. Combination of ≥3 symptoms outlined above

Strongly Disagree 1	2	3	4	5	6	7	8	Strongly Agree 9
★	★	★	★	★	★	★	★	★

* 235. Radiology suggestive of NTM

Strongly Disagree 1	2	3	4	5	6	7	8	Strongly Agree 9
★	★	★	★	★	★	★	★	★

* 236. Before starting macrolides

Strongly Disagree 1	2	3	4	5	6	7	8	Strongly Agree 9
☆	☆	☆	☆	☆	☆	☆	☆	☆

* 237. In receipt of macrolides

Strongly Disagree 1	2	3	4	5	6	7	8	Strongly Agree 9
☆	☆	☆	☆	☆	☆	☆	☆	☆

* 238. In receipt of inhaled steroids

Strongly Disagree 1	2	3	4	5	6	7	8	Strongly Agree 9
☆	☆	☆	☆	☆	☆	☆	☆	☆

* 239. In receipt of immunosuppressive/immune disorder drugs

Strongly Disagree 1	2	3	4	5	6	7	8	Strongly Agree 9
☆	☆	☆	☆	☆	☆	☆	☆	☆

CVD

All adults with CVD who have one or more of the following symptoms should be tested for NTM

* 240. No symptoms

Strongly disagree 1	2	3	4	5	6	7	8	Strongly Agree 9
★	★	★	★	★	★	★	★	★

* 241. Persistent cough

Strongly Disagree 1	2	3	4	5	6	7	8	Strongly Agree 9
★	★	★	★	★	★	★	★	★

* 242. Unexplained weight loss

Strongly Disagree 1	2	3	4	5	6	7	8	Strongly Agree 9
★	★	★	★	★	★	★	★	★

* 243. Night sweats

Strongly Disagree 1	2	3	4	5	6	7	8	Strongly Agree 9
★	★	★	★	★	★	★	★	★

* 244. Haemoptysis

Strongly Disagree 1	2	3	4	5	6	7	8	Strongly Agree 9
★	★	★	★	★	★	★	★	★

* 245. Persistent sputum production

Strongly Disagree 1	2	3	4	5	6	7	8	Strongly Agree 9
★	★	★	★	★	★	★	★	★

* 246. Persistent fatigue

Strongly Disagree 1	2	3	4	5	6	7	8	Strongly Agree 9
☆	☆	☆	☆	☆	☆	☆	☆	☆

* 247. Recurrent respiratory infection

Strongly Disagree 1	2	3	4	5	6	7	8	Strongly Agree 9
☆	☆	☆	☆	☆	☆	☆	☆	☆

* 248. Persistent unexplained fever

Strongly Disagree 1	2	3	4	5	6	7	8	Strongly Agree 9
☆	☆	☆	☆	☆	☆	☆	☆	☆

* 249. Persistent shortness of breath

Strongly Disagree 1	2	3	4	5	6	7	8	Strongly Agree 9
☆	☆	☆	☆	☆	☆	☆	☆	☆

* 250. Combination of ≥2 symptoms outlined above

Strongly Disagree 1	2	3	4	5	6	7	8	Strongly Agree 9
☆	☆	☆	☆	☆	☆	☆	☆	☆

* 251. Combination of ≥3 symptoms outlined above

Strongly Disagree 1	2	3	4	5	6	7	8	Strongly Agree 9
☆	☆	☆	☆	☆	☆	☆	☆	☆

* 252. Radiology suggestive of NTM

Strongly Disagree 1	2	3	4	5	6	7	8	Strongly Agree 9
☆	☆	☆	☆	☆	☆	☆	☆	☆

* 253. Before starting macrolides

Strongly Disagree 1	2	3	4	5	6	7	8	Strongly Agree 9
☆	☆	☆	☆	☆	☆	☆	☆	☆

* 254. In receipt of macrolides

Strongly Disagree 1	2	3	4	5	6	7	8	Strongly Agree 9
☆	☆	☆	☆	☆	☆	☆	☆	☆

* 255. In receipt of inhaled steroids

Strongly Disagree 1	2	3	4	5	6	7	8	Strongly Agree 9
☆	☆	☆	☆	☆	☆	☆	☆	☆

* 256. In receipt of immunosuppressive/immune disorder drugs

Strongly Disagree 1	2	3	4	5	6	7	8	Strongly Agree 9
☆	☆	☆	☆	☆	☆	☆	☆	☆

Active/ongoing cancer

All adults with active/ongoing cancer who have one or more of the following symptoms should be tested for NTM

* 257. No symptoms

Strongly disagree 1	2	3	4	5	6	7	8	Strongly Agree 9
★	★	★	★	★	★	★	★	★

* 258. Persistent cough

Strongly Disagree 1	2	3	4	5	6	7	8	Strongly Agree 9
★	★	★	★	★	★	★	★	★

* 259. Unexplained weight loss

Strongly Disagree 1	2	3	4	5	6	7	8	Strongly Agree 9
★	★	★	★	★	★	★	★	★

* 260. Night sweats

Strongly Disagree 1	2	3	4	5	6	7	8	Strongly Agree 9
★	★	★	★	★	★	★	★	★

* 261. Haemoptysis

Strongly Disagree 1	2	3	4	5	6	7	8	Strongly Agree 9
★	★	★	★	★	★	★	★	★

* 262. Persistent sputum production

Strongly Disagree 1	2	3	4	5	6	7	8	Strongly Agree 9
★	★	★	★	★	★	★	★	★

* 263. Persistent fatigue

Strongly Disagree 1	2	3	4	5	6	7	8	Strongly Agree 9
☆	☆	☆	☆	☆	☆	☆	☆	☆

* 264. Recurrent respiratory infection

Strongly Disagree 1	2	3	4	5	6	7	8	Strongly Agree 9
☆	☆	☆	☆	☆	☆	☆	☆	☆

* 265. Persistent unexplained fever

Strongly Disagree 1	2	3	4	5	6	7	8	Strongly Agree 9
☆	☆	☆	☆	☆	☆	☆	☆	☆

* 266. Persistent shortness of breath

Strongly Disagree 1	2	3	4	5	6	7	8	Strongly Agree 9
☆	☆	☆	☆	☆	☆	☆	☆	☆

* 267. Combination of ≥2 symptoms outlined above

Strongly Disagree 1	2	3	4	5	6	7	8	Strongly Agree 9
☆	☆	☆	☆	☆	☆	☆	☆	☆

* 268. Combination of ≥3 symptoms outlined above

Strongly Disagree 1	2	3	4	5	6	7	8	Strongly Agree 9
☆	☆	☆	☆	☆	☆	☆	☆	☆

* 269. Radiology suggestive of NTM

Strongly Disagree 1	2	3	4	5	6	7	8	Strongly Agree 9
☆	☆	☆	☆	☆	☆	☆	☆	☆

* 270. Before starting macrolides

Strongly Disagree 1	2	3	4	5	6	7	8	Strongly Agree 9
☆	☆	☆	☆	☆	☆	☆	☆	☆

* 271. In receipt of macrolides

Strongly Disagree 1	2	3	4	5	6	7	8	Strongly Agree 9
☆	☆	☆	☆	☆	☆	☆	☆	☆

* 272. In receipt of inhaled steroids

Strongly Disagree 1	2	3	4	5	6	7	8	Strongly Agree 9
☆	☆	☆	☆	☆	☆	☆	☆	☆

* 273. In receipt of immunosuppressive/immune disorder drugs

Strongly Disagree 1	2	3	4	5	6	7	8	Strongly Agree 9
☆	☆	☆	☆	☆	☆	☆	☆	☆

GERD

All adults with GERD who have one or more of the following symptoms should be tested for NTM

* 274. No symptoms

Strongly disagree 1	2	3	4	5	6	7	8	Strongly Agree 9
★	★	★	★	★	★	★	★	★

* 275. Persistent cough

Strongly Disagree 1	2	3	4	5	6	7	8	Strongly Agree 9
★	★	★	★	★	★	★	★	★

* 276. Unexplained weight loss

Strongly Disagree 1	2	3	4	5	6	7	8	Strongly Agree 9
★	★	★	★	★	★	★	★	★

* 277. Night sweats

Strongly Disagree 1	2	3	4	5	6	7	8	Strongly Agree 9
★	★	★	★	★	★	★	★	★

* 278. Haemoptysis

Strongly Disagree 1	2	3	4	5	6	7	8	Strongly Agree 9
★	★	★	★	★	★	★	★	★

* 279. Persistent sputum production

Strongly Disagree 1	2	3	4	5	6	7	8	Strongly Agree 9
★	★	★	★	★	★	★	★	★

* 280. Persistent fatigue

Strongly Disagree 1	2	3	4	5	6	7	8	Strongly Agree 9
☆	☆	☆	☆	☆	☆	☆	☆	☆

* 281. Recurrent respiratory infection

Strongly Disagree 1	2	3	4	5	6	7	8	Strongly Agree 9
☆	☆	☆	☆	☆	☆	☆	☆	☆

* 282. Persistent unexplained fever

Strongly Disagree 1	2	3	4	5	6	7	8	Strongly Agree 9
☆	☆	☆	☆	☆	☆	☆	☆	☆

* 283. Persistent shortness of breath

Strongly Disagree 1	2	3	4	5	6	7	8	Strongly Agree 9
☆	☆	☆	☆	☆	☆	☆	☆	☆

* 284. Combination of ≥2 symptoms outlined above

Strongly Disagree 1	2	3	4	5	6	7	8	Strongly Agree 9
☆	☆	☆	☆	☆	☆	☆	☆	☆

* 285. Combination of ≥3 symptoms outlined above

Strongly Disagree 1	2	3	4	5	6	7	8	Strongly Agree 9
☆	☆	☆	☆	☆	☆	☆	☆	☆

* 286. Radiology suggestive of NTM

Strongly Disagree 1	2	3	4	5	6	7	8	Strongly Agree 9
☆	☆	☆	☆	☆	☆	☆	☆	☆

* 287. Before starting macrolides

Strongly Disagree 1	2	3	4	5	6	7	8	Strongly Agree 9
☆	☆	☆	☆	☆	☆	☆	☆	☆

* 288. In receipt of macrolides

Strongly Disagree 1	2	3	4	5	6	7	8	Strongly Agree 9
☆	☆	☆	☆	☆	☆	☆	☆	☆

* 289. In receipt of inhaled steroids

Strongly Disagree 1	2	3	4	5	6	7	8	Strongly Agree 9
☆	☆	☆	☆	☆	☆	☆	☆	☆

* 290. In receipt of immunosuppressive/immune disorder drugs

Strongly Disagree 1	2	3	4	5	6	7	8	Strongly Agree 9
☆	☆	☆	☆	☆	☆	☆	☆	☆

Delphi process – Round 3 Voting Sheet

Delphi Round 3

Using the questions below in particular patient types, please score how likely or not you are to test for NTM in patients presenting with symptoms or clinical scenarios.

Score 7-9 means high agreement that NTM should be tested for
Score 4-6 means equivocal agreement, testing in some circumstances
Score 1-3 means testing is not suggested/required as the likelihood of NTM infection is low

		**Would you test for NTM in patients with no known respiratory disease or with no previous investigations for suspected respiratory disease if the following symptoms or clinical scenarios were present?**	**Would you test for NTM in patients with a previous history of NTM if the following symptoms or clinical scenarios were present?** ***(This question assumes that your index of suspicion for NTM would be high for subsequent infection)***
		Score (please add numerical score 1-9)	***Score (please add numerical score 1-9)***
1	No symptoms		
2	Persistent cough		
3	Unexplained weight loss		
4	Night sweats		
5	Haemoptysis		
6	Persistent sputum production		
7	Persistent fatigue		
8	Recurrent respiratory infection		
9	Persistent unexplained fever		
10	Persistent shortness of breath		
11	Combination of ≥2 symptoms outlined above		
12	Combination of ≥3 symptoms outlined above		

13	Radiology suggestive of NTM		
14	Before starting long-term macrolides for a non-respiratory cause or non-respiratory condition		
15	In receipt of long-term macrolides for a non-respiratory cause or non-respiratory condition		
16	In receipt of inhaled steroids		
17	In receipt of immunosuppressive/immune disorder drugs for non-respiratory reasons e.g. biologics		

Summary sheet - mean scores by comorbidity and symptom

Would you test for NTM in these patients if the following clinical scenarios were present?

		No known respiratory disease or with no previous investigations for suspected respiratory disease	Bronchiectasis	Asthma	CF	COPD	History of TB	ILD	Fungal lung disease	Immunosuppressed pts	Post-transplant patients	Immune disorders	Rheumatoid arthritis	Renal disease	Diabetes	CVD	Cancer	GERD
1	No symptoms																	
2	Persistent cough																	
3	Unexplained weight loss																	
4	Night sweats																	
5	Haemoptysis																	
6	Persistent sputum production																	
7	Persistent fatigue																	
8	Recurrent respiratory infection																	
9	Persistent unexplained fever																	
10	Persistent shortness of breath																	
11	Combination of ≥2 symptoms outlined above																	
12	Combination of ≥3 symptoms outlined above																	
13	Radiology suggestive of NTM																	
14	Before starting long-term macrolides for a non-respiratory cause or non-respiratory condition																	
15	In receipt of long-term macrolides for a non-respiratory cause or non-respiratory condition																	
16	In receipt of inhaled steroids																	
17	In receipt of immunosuppressive/immune disorder drugs for non-respiratory reasons e.g. biologics																	

In the meeting, it was agreed that there is a need to clarify scoring with respect to the use of medications in non-respiratory conditions. Please complete the white cells only with your score.

Please score in these patient types and clinical scenarios how likely or not you are to test for NTM.

Score 7-9 means high agreement that NTM should be tested for
Score 4-6 means equivocal agreement, perhaps testing in some circumstances or lack of evidence to consider testing
Score 1-3 means testing is not suggested/required as the likelihood of NTM infection is low
